# Association of Pregnancy and Insurance Status With Treatment Access for Opioid Use Disorder

**DOI:** 10.1001/jamanetworkopen.2020.13456

**Published:** 2020-08-14

**Authors:** Stephen W. Patrick, Michael R. Richards, William D. Dupont, Elizabeth McNeer, Melinda B. Buntin, Peter R. Martin, Matthew M. Davis, Corey S. Davis, Katherine E. Hartmann, Ashley A. Leech, Kim S. Lovell, Bradley D. Stein, William O. Cooper

**Affiliations:** 1 Vanderbilt Center for Child Health Policy, Vanderbilt University Medical Center, Nashville, Tennessee; 2 Department of Pediatrics, Vanderbilt University Medical Center, Nashville, Tennessee; 3 Department of Health Policy, Vanderbilt University Medical Center, Nashville, Tennessee; 4 Department of Economics, Baylor University, Waco, Texas; 5 Department of Biostatistics, Vanderbilt University Medical Center, Nashville, Tennessee; 6 Department of Psychiatry and Behavioral Sciences, Vanderbilt University Medical Center, Nashville, Tennessee; 7 Department of Pharmacology, Vanderbilt University Medical Center, Nashville, Tennessee; 8 Department of Pediatrics, Ann & Robert H. Lurie Children’s Hospital and Northwestern University Feinberg School of Medicine, Chicago, Illinois; 9 Network for Public Health Law, Los Angeles, California; 10 Department of Obstetrics and Gynecology, Vanderbilt University Medical Center, Nashville, Tennessee; 11 RAND Corporation, Pittsburgh, Pennsylvania; 12 Department of Psychiatry, University of Pittsburgh School of Medicine, Pittsburgh, Pennsylvania

## Abstract

**Importance:**

Medications for opioid use disorder, including buprenorphine hydrochloride and methadone hydrochloride, are highly effective at improving outcomes for individuals with the disorder. For pregnant women, use of these medications also improves pregnancy outcomes, including the risk of preterm birth. Despite the known benefits of medications for opioid use disorder, many pregnant and nonpregnant women with the disorder are not receiving them.

**Objective:**

To determine whether pregnancy and insurance status are associated with a woman’s ability to obtain an appointment with an opioid use disorder treatment clinician.

**Design, Setting, and Participants:**

In this cross-sectional study with random assignment of clinicians and simulated-patient callers (performed in “secret shopper” format), outpatient clinics that provide buprenorphine and methadone were randomly selected from publicly available treatment lists in 10 US states (selected for variability in opioid-related outcomes and policies) from March 7 to September 5, 2019. Pregnant vs nonpregnant woman and private vs public insurance assigned randomly to callers to create unique patient profiles. Simulated patients called the clinics posing as pregnant or nonpregnant women to obtain an initial appointment with a clinician.

**Main Outcomes and Measures:**

Appointment scheduling, wait time, and out-of-pocket costs.

**Results:**

A total of 10 871 unique patient profiles were assigned to 6324 clinicians. Among all women, 2312 of 3420 (67.6%) received an appointment with a clinician who prescribed buprenorphine, with lower rates among pregnant vs nonpregnant callers (1055 of 1718 [61.4%] vs 1257 of 1702 [73.9%]; relative risk, 0.83; 95% CI, 0.79-0.87). For clinicians who prescribed methadone, there was no difference in appointment access for pregnant vs nonpregnant callers (240 of 271 [88.6%] vs 237 of 265 [89.4%]; relative risk, 0.99; 95% CI, 0.93-1.05). Insurance was frequently not accepted, with 894 of 3420 buprenorphine-waivered prescribers (26.1%) and 174 of 536 opioid treatment programs (32.5%) granting appointments only when patients agreed to pay cash. Median wait times did not differ between pregnant and nonpregnant callers among buprenorphine prescribers (3 days [interquartile range, 1-7 days] vs 3 days [interquartile range, 1-7 days]; *P* = .43) but did differ among methadone prescribers (1 day [interquartile range, 1-4 days] vs 2 days [interquartile range, 1-6 days]; *P* = .049). For patients agreeing to pay cash, the median out-of-pocket costs for initial appointments were $250 (interquartile range, $155-$300) at buprenorphine prescribers and $34 (interquartile range, $15-$120) at methadone prescribers.

**Conclusions and Relevance:**

In this cross-sectional study with random assignment of clinicians and simulated-patient callers, many women, especially pregnant women, faced barriers to accessing treatment. Given the high out-of-pocket costs and lack of acceptance of insurance among many clinicians, access to affordable opioid use disorder treatment is a significant concern.

## Key Points

**Question:**

Are pregnancy and insurance status associated with a woman’s ability to obtain an appointment with an opioid use disorder treatment clinician?

**Findings:**

In this cross-sectional study with random assignment of clinicians and simulated-patient callers, callers representing pregnant women were less likely than callers representing nonpregnant women to be granted an appointment with an opioid use disorder treatment clinician (61% vs 74%). There were substantial barriers for both pregnant and nonpregnant women attempting to gain access to treatment, including a large portion of clinicians who did not accept insurance and required cash payment for an appointment.

**Meaning:**

These findings suggest that pregnant and nonpregnant women face substantial barriers in obtaining appointments with an opioid use disorder treatment clinician.

## Teaser

This cross-sectional study examines whether pregnancy and insurance status are associated with a woman’s ability to obtain an appointment with an opioid use disorder treatment clinician.

## Introduction

Opioid use during pregnancy,^1,2^ diagnoses of opioid use disorder (OUD) among pregnant women,^3,4,5^ and neonatal complications from in utero opioid exposure have increased substantially during the past 2 decades.^3,5,6,7,8^ Untreated OUD among pregnant women is associated with adverse pregnancy outcomes, including overdose death and preterm birth.^9^ Treatment with medications for OUD (MOUDs) is associated with a reduction in the risk of these adverse outcomes.^9,10^ Methadone hydrochloride, a full μ-opioid receptor agonist, and buprenorphine hydrochloride, a partial μ-opioid receptor agonist and κ-opioid receptor antagonist, are medications for OUD recommended during pregnancy.^11^ Methadone for OUD treatment is dispensed only in federally regulated opioid treatment programs (OTPs), whereas buprenorphine is frequently prescribed in outpatient settings by clinicians with a federal waiver to prescribe the medication and can be obtained from an OTP.

Despite evidence that MOUDs are effective in mitigating adverse pregnancy outcomes, many pregnant women with OUD are not receiving them.^12^ A recent National Academy of Medicine report stated that confronting barriers to access to MOUDs, especially for vulnerable populations such as pregnant women, is essential to reduce opioid-related harm.^13^ Understanding real-world barriers to access to MOUDs is vital to creating systems of care that improve access for pregnant women; however, empirical studies of barriers to access to MOUDs for pregnant women are sparse, to our knowledge.^14,15^ To better understand patient barriers to access to medical care, the US Department of Health and Human Services has recommended a field experiment (ie, “secret shopper”) study design.^16,17,18,19,20^ In this study, we conducted a cross-sectional study with random assignment of clinicians and simulated-patient callers to obtain unbiased estimates of differences in treatment access for pregnant women vs nonpregnant women of reproductive age in OTPs and from buprenorphine-waivered prescribers.

## Methods

The study was conducted from March 7 to September 5, 2019, among 10 states (Florida, Kentucky, Massachusetts, Michigan, Missouri, North Carolina, Tennessee, Virginia, Washington, and West Virginia) selected for their broad range of opioid-related complications (eg, opioid-related overdose deaths)^21^ and state policies (eg, Medicaid expansion under the Patient Protection and Affordable Care Act).^22^ Based on pilot data,^23^ we hypothesized that pregnant women would have more difficulty than nonpregnant women in obtaining treatment access, that there would be little difference in treatment access between women covered by public insurance and women covered by private insurance, and that many clinicians would require cash payment rather than accepting insurance.

Our primary outcome was the ability of simulated patients who called (hereafter referred to as callers) to obtain an initial appointment with an OTP or buprenorphine-waivered prescriber. Our secondary outcomes were wait times and stated out-of-pocket costs for patients with clinicians who offered cash-only appointments. Script development and field testing occurred in nonsample states (Arkansas, California, Connecticut, New Mexico, and New York). The University of Chicago Survey Lab provided consultation and assistance with the script development and testing and conducted calls for the field experiment. This study was deemed exempt under CFR 46.101, b(2) from human participants review by the University of Chicago and Vanderbilt University Medical Center institutional review boards. This study adhered to the relevant sections of the Consolidated Standards of Reporting Trials (CONSORT) reporting guideline for studies using randomized assignment

### Data Sources

To simulate the real-world experience of a patient attempting to access MOUDs, we obtained contact information for OTPs and buprenorphine-waivered prescribers from public lists maintained by the Substance Abuse and Mental Health Services Administration (SAMHSA). We obtained data for buprenorphine-waivered prescribers from the Buprenorphine Practitioner Locator^24^ and for OTPs from the Opioid Treatment Program Directory,^25^ downloaded in December 2018. A county-level market analysis of private and Medicaid managed-care insurance plans was performed using 2018 data from the Decision Resources Group.^26^ Callers were assigned the most common private or Medicaid managed-care insurer based on insurer market share within the county of their randomization assignment.

### Script Development

In the initial phase, we developed a script for women with OUD by interviewing treatment clinician staff and calling several buprenorphine-waivered prescribers and OTPs to better understand what information would be needed to make an appointment to obtain MOUD treatment. We developed and refined the script (eAppendix 1 in the Supplement) to create a scenario that demonstrated the need for treatment (ie, active opioid use) without the need for emergency care (ie, suicidal ideation or intent). In January 2019, we conducted a pilot study of 191 OTPs and buprenorphine-waivered prescribers (outside of the experimental sample), in which callers posing as either a pregnant woman with OUD or a nonpregnant woman of reproductive age with OUD attempted to make an appointment to obtain MOUD treatment. During the pilot study, we progressively refined the script to minimize call time and create plausible responses for common questions.

### Randomization Process

Between March 7 and September 5, 2019, we conducted the randomized field experiment. Only unique practices were called (ie, a clinic with >1 clinician was sampled only once). Given that patient characteristics may be associated with a patient’s ability to obtain an appointment,^27^ 9 women were hired as callers, representing a spectrum of White, Hispanic, and African American female vocal features across an age range of 25 to 30 years. Callers used a computer-assisted telephone interviewing system that displayed the script for the specific randomization assignment to facilitate data collection. Caller scripts assigned to pregnant women differed from nonpregnant women by adding “I’m 4 months pregnant” when requesting an appointment. Patient names were assigned using the Social Security Administration’s website, which provides the most common first names by sex and birth decade, and using the 2000 census to obtain the top 1000 names by race/ethnicity. Patient addresses and telephone numbers were obtained from vacant addresses and disconnected telephone numbers in the clinician’s geographical area. Email addresses were frequently requested during pilot calls; therefore, each caller was assigned an email composed of the patient’s first name, a random letter, their simulated patient’s surname, and 3 random digits. Appointments were canceled prior to ending the call. Callers completed a standardized data collection tool during the call (eAppendix 2 in the Supplement).

We created randomization schemes for buprenorphine-waivered prescribers and OTPs using a blocked randomized design that balanced patient characteristics and limited the number of calls to individual clinicians. Each caller was equally likely to be assigned as a pregnant or nonpregnant patient. For buprenorphine-waivered prescribers, 4 patient profiles (pregnant or not pregnant with Medicaid or private insurance) were randomly assigned. We used permuted block randomization to assign the same number of profiles to groups of clinicians, allowing no more than 3 calls per clinician (eAppendix 3 in the Supplement).

### Statistical Analysis

Pearson contingency table χ^2^ statistics were used for simple comparisons of categorical variables between pregnant and nonpregnant callers. The success rate was calculated for the pregnant and nonpregnant groups and for the Medicaid and private insurance groups by state, and Wilson 95% CIs were computed. Relative risks of obtaining an appointment for pregnant vs nonpregnant callers were obtained from incidence rate ratios. These statistics, together with 95% CIs and *P* values, were derived using Wald statistics. To test whether appointment access varied by state, we performed likelihood ratio tests between an intercept-only model and a model including state as a predictor. We used χ^2^ tests to test whether there were differences in insurance acceptance. For the secondary outcomes of cost and wait time, we used Wilcoxon rank sum tests to test for differences between the pregnant and nonpregnant groups. We conducted a supplemental analysis using a mixed-effects logistic regression model with random intercepts for clinicians to test the robustness of our results (eTable 1 in the Supplement). All *P* values were from 2-sided tests, and results were deemed statistically significant at *P* < .05. Analyses were performed using R, version 3.6.3 (R Core Team) (eAppendix 4 in the Supplement).

## Results

There were no significant differences between represented patients’ pregnancy status, insurance type, age, race/ethnicity, or state of residence among callers who successfully spoke with a clinician (Table 1; eTable 2 in the Supplement). A total of 28 651 calls were made by 10 871 callers (10 117 to buprenorphine-waivered prescribers and 754 to OTPs) to 6324 clinicians (5944 buprenorphine-waivered prescribers and 380 OTPs). Overall, 1718 pregnant callers (853 public insurance and 865 private insurance) and 1702 nonpregnant callers (860 public insurance and 842 private insurance) reached the practice of a buprenorphine-waivered prescriber; 271 pregnant callers and 265 nonpregnant callers (all public insurance) reached an OTP. The success rate for reaching a practice was 33.8% (3420 of 10 117) for buprenorphine-waivered prescribers and 71.1% (536 of 754) for OTPs.

**Table 1.** Characteristics of Pregnant and Nonpregnant Callers Attempting to Access Treatment

Characteristic	Buprenorphine-waivered prescribers			Opioid treatment programs		
	Nonpregnant (n = 1702)	Pregnant (n = 1718)	*P* value	Nonpregnant (n = 265)	Pregnant (n = 271)	*P* value
Insurance						
Public	860 (50.5)	853 (49.7)	.61	265 (100)	271 (100)	NA
Private	842 (49.5)	865 (50.3)		NA	NA	
Race/ethnicity						
Black	595 (35.0)	642 (37.4)	.14	104 (39.2)	90 (33.2)	.24
White	181 (10.6)	154 (9.0)		15 (5.7)	22 (8.1)	
Hispanic	926 (54.4)	922 (53.7)		146 (55.1)	159 (58.7)	
Age, y						
25	248 (14.6)	289 (16.8)	.56	45 (17.0)	48 (17.7)	.30
26	288 (16.9)	268 (15.6)		46 (17.4)	37 (13.7)	
27	280 (16.5)	280 (16.3)		37 (14.0)	53 (19.6)	
28	285 (16.7)	279 (16.2)		41 (15.5)	51 (18.8)	
29	303 (17.8)	299 (17.4)		49 (18.5)	42 (15.5)	
30	298 (17.5)	303 (17.6)		47 (17.7)	40 (14.8)	
State						
Florida	200 (11.8)	173 (10.1)	.94	46 (17.4)	51 (18.8)	>.99
Kentucky	178 (10.5)	187 (10.9)		20 (7.5)	20 (7.4)	
Massachusetts	120 (7.1)	113 (6.6)		45 (17.0)	38 (14.0)	
Michigan	196 (11.5)	198 (11.5)		19 (7.2)	23 (8.5)	
Missouri	179 (10.5)	183 (10.7)		12 (4.5)	13 (4.8)	
North Carolina	198 (11.6)	199 (11.6)		56 (21.1)	59 (21.8)	
Tennessee	207 (12.2)	215 (12.5)		13 (4.9)	13 (4.8)	
Virginia	152 (8.9)	168 (9.8)		31 (11.7)	28 (10.3)	
Washington	170 (10.0)	178 (10.4)		16 (6.0)	18 (6.6)	
West Virginia	102 (6.0)	104 (6.1)		7 (2.6)	8 (3.0)	

Abbreviation: NA, not applicable.

Among both buprenorphine-waivered prescribers and OTPs, the most common reasons for the inability to reach a clinician were 5 or more attempts made without an answer (2409 of 10 117 [23.8%] for buprenorphine prescribers and 62 of 754 [8.2%] for OTPs) and reaching a medical practice that does not provide treatment of OUD (1999 of 10 117 [19.8%] for buprenorphine prescribers and 25 of 754 [3.3%] for OTPs) (eTable 3, eFigure 1, and eFigure 2 in the Supplement). The median number of calls were 2 (interquartile range [IQR], 1-5) for buprenorphine-waivered prescribers and 2 (IQR, 1-3) for OTPs to speak with a member of the clinic staff who could schedule an appointment (eTable 4 in the Supplement).

**Figure 1.**
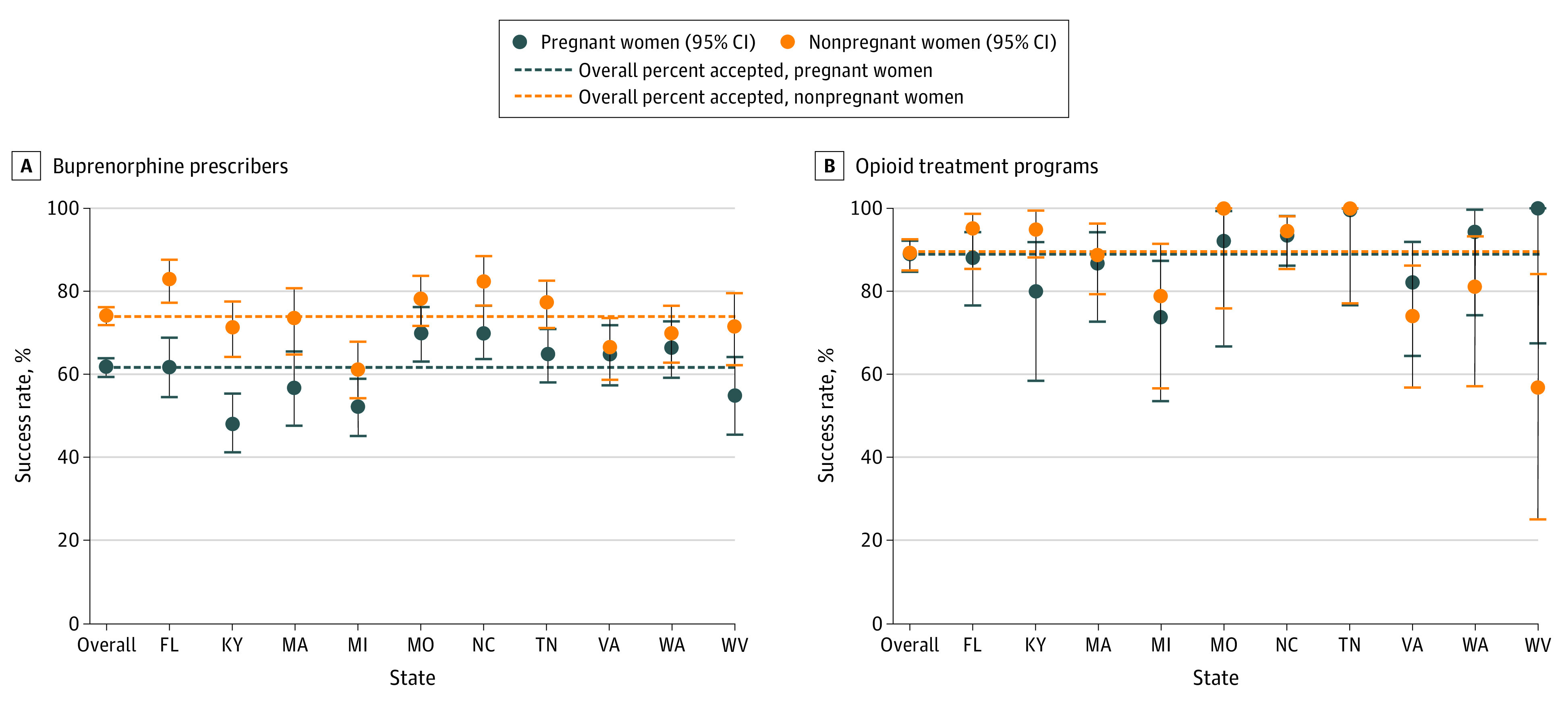
Ability of Pregnant and Nonpregnant Callers to Obtain an Appointment for Treatment Among Buprenorphine-Waivered Prescribers and Opioid Treatment Programs Ability to obtain an appointment with insurance or cash payment.

Among all women, 2312 of 3420 (67.6%) received an appointment with a buprenorphine clinician. Pregnant callers were less likely than nonpregnant callers to be given an appointment with a buprenorphine-waivered prescriber (1055 of 1718 [61.4%] vs 1257 of 1702 [73.9%]; relative risk, 0.83; 95% CI, 0.79-0.87). There was substantial variability among states in appointment access with buprenorphine-waivered prescribers, ranging from 48.1% (90 of 187) in Kentucky to 70.4% (140 of 199) in North Carolina (*P* < .001) for pregnant callers and ranging from 61.2% (120 of 196) in Michigan to 83.0% (166 of 200) in Florida (*P* < .001) for nonpregnant callers. Although some states showed substantial differences in the ability of pregnant and nonpregnant callers to obtain appointments (eg, Kentucky: 90 of 187 [48.1%] vs 127 of 178 [71.3%]), there was no difference in other states (eg, Virginia: 109 of 168 [64.9%] vs 101 of 152 [66.4%]). In contrast, there was no overall difference in appointment access to OTPs for pregnant vs nonpregnant callers (240 of 271 [88.6%] vs 237 of 265 [89.4%]; relative risk, 0.99; 95% CI, 0.93-1.05) and no significant differences between a pregnant and a nonpregnant caller’s ability to obtain an OTP appointment within any state (Figure 1; eTables 5, 6, and 7 in the Supplement).

With both buprenorphine-waivered prescribers and OTPs, insurance was associated with appointment access. Nonpregnant callers with Medicaid were less likely than nonpregnant callers with private insurance to be granted an appointment with buprenorphine-waivered prescribers (347 of 860 [40.3%] vs 414 of 842 [49.2%]; *P* < .001) (Table 2); however, there were no differences in insurance acceptance among pregnant women. Among OTPs, 155 of 271 pregnant callers (57.2%) and 148 of 265 nonpregnant callers (55.8%) with Medicaid were granted appointments (Table 2).

**Table 2.** Ability to Obtain an Appointment Among Pregnant and Nonpregnant Callers Among Buprenorphine-Waivered Prescribers and Opioid Treatment Programs by Insurance Type

Characteristic	No./total No. (%)		*P* value
	Medicaid	Private	
**Buprenorphine-waivered prescribers**^**a**^			
Pregnant			
Accepted	310/853 (36.3)	347/865 (40.1)	.11
Rejected, cash accepted	207/853 (24.3)	191/865 (22.1)	.28
Unable to make appointment	336/853 (39.4)	327/865 (37.8)	.50
Nonpregnant			
Accepted	347/860 (40.3)	414/842 (49.2)	<.001
Rejected, cash accepted	259/860 (30.1)	237/842 (28.1)	.37
Unable to make appointment	254/860 (29.5)	191/842 (22.7)	<.001
**Opioid treatment programs**^**b**^			
Pregnant			
Accepted	155/271 (57.2)	NA	NA
Rejected, cash accepted	85/271 (31.4)	NA	NA
Unable to make appointment	31/271 (11.4)	NA	NA
Nonpregnant			
Accepted	148/265 (55.8)	NA	NA
Rejected, cash accepted	89/265 (33.6)	NA	NA
Unable to make appointment	28/265 (10.6)	NA	NA

Abbreviation: NA, not applicable.

^a^ Patients randomized to pregnant with Medicaid, nonpregnant with Medicaid, pregnant with private insurance, and nonpregnant with private insurance. If appointments were refused with initial insurance, patients offered to pay cash.

^b^ Patients randomized to pregnant or nonpregnant with Medicaid. If appointments were refused with initial insurance, patients offered to pay cash.

For both buprenorphine-waivered prescribers and OTPs, appointments were offered only when callers agreed to pay cash at 894 of 3420 buprenorphine-waivered prescribers (26.1%) and 174 of 536 OTPs (32.5%). For callers agreeing to pay cash for treatment, the median out-of-pocket costs for initial appointments were $250 (IQR, $155-$300) at buprenorphine-waivered prescribers and $34 (IQR, $15-$120) at OTPs (eTable 8 in the Supplement). There was no significant difference between median wait times for pregnant vs nonpregnant callers getting appointments for treatment at buprenorphine-waivered prescribers (3 days [IQR, 1-7 days] vs 3 days [IQR, 1-7 days]; *P* = .43). However, there were small but statistically significant differences for pregnant vs nonpregnant callers in wait times for appointments with OTPs (1 day [IQR, 1-4 days] vs 2 days [IQR, 1-6 days]; *P* = .049) (eTable 9 in the Supplement).

**Figure 2.**
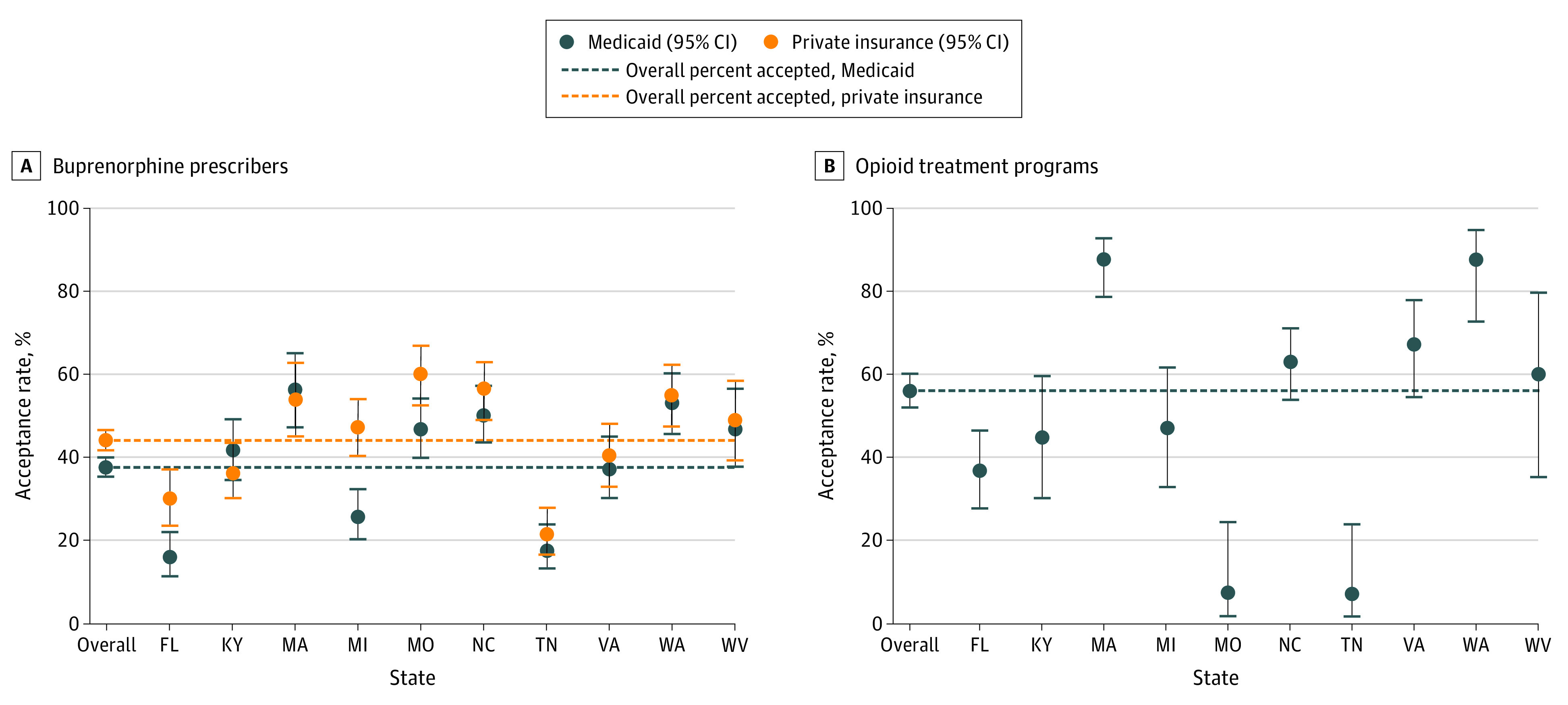
Acceptance of Medicaid or Private Insurance for Treatment Among Buprenorphine-Waivered Prescribers and Acceptance of Medicaid for Treatment Among Opioid Treatment Programs

There were substantial differences across states in acceptance of insurance for treatment at buprenorphine-waivered prescribers and OTPs. Among buprenorphine-waivered prescribers, Medicaid acceptance ranged from 32 of 193 (16.6%) in Florida to 65 of 114 (57.0%) in Massachusetts (*P* < .001). Acceptance of private insurance ranged from 48 of 216 (22.2%) in Tennessee to 107 of 177 (60.5%) in Missouri (*P* < .001). Among OTPs, acceptance of Medicaid ranged from 2 of 26 (7.7%) in Tennessee to 73 of 83 (88.0%) in Massachusetts (*P* < .001) (Figure 2).

## Discussion

In this cross-sectional study with random assignment of clinicians and simulated-patient callers, we found many barriers to women successfully accessing MOUDs; these barriers were more substantial for pregnant women. Pregnant callers were 17% less likely to be granted an appointment with a buprenorphine-waivered prescriber than nonpregnant women, and a large subgroup of prescribers did not accept any type of insurance. As highlighted by the National Academy of Medicine^13^ and the US Surgeon General,^28^ improving access to MOUDs for pregnant women with OUD is a public health priority. Despite this fact, our study suggests that a significant portion of pregnant women cannot easily access MOUD treatment—even when a prescriber is nearby.

Buprenorphine and methadone are critical components in the treatment of pregnant women with OUD.^29^ Medications for OUD are associated with a reduction in relapse risk,^30,31,32,33^ HIV risk,^30,34^ criminal behavior,^32^ and risk of overdose death^35^ and with improvement in treatment retention^30^ and birth weight.^36^ Because of differences in drug mechanisms and treatment strategies, some women have better outcomes with one medication compared with another. For example, treatment with methadone requires daily visits to OTPs that may improve treatment retention,^29^ whereas the flexibility of buprenorphine treatment with regard to visits may improve other outcomes, such as maintaining employment. For these reasons, SAMHSA guidelines suggest that pregnant women should have access to both medications.^11^ Ideally, pregnant women would have access to comprehensive programs that provide prenatal care, treat additional psychiatric and medical comorbidities, and provide counseling and MOUDs.^37^

We found that pregnant callers attempting to access treatment at OTPs that provide methadone were more likely to obtain an appointment than at buprenorphine-waivered prescribers; however, OTPs are less common in the United States than buprenorphine-waivered prescribers. For example, at the time of our study, there were only 9 unique OTPs in West Virginia compared with 222 buprenorphine-waivered prescribers. Furthermore, much of the recent expansion of MOUDs has focused on expanding the number of buprenorphine-waivered prescribers rather than OTPs.^38^

One possible explanation for our findings is that few women’s health clinicians have received waivers to prescribe buprenorphine. Less than 1% of obstetricians have received waivers,^39^ while nearly 4% of family medicine physicians in the United States have received waivers.^39^ The willingness and comfort of family medicine physicians and other women’s health clinicians to provide pregnant women MOUD treatment is not known. Furthermore, many waivered prescribers do not actively prescribe buprenorphine.^40^ We found that 19.8% of clinicians (1999 of 10 117) who appear on SAMHSA’s list of waivered clinicians called do not prescribe buprenorphine, which suggests that they may have obtained waiver status but are not using it.

Similar to a recent study,^41^ we also found that the publicly available treatment locator published by SAMHSA is not a reliable source of information for waivered prescribers; nearly one-fourth of telephone numbers were called at least 5 times with no answer, and nearly 20% of the medical offices that we successfully contacted did not provide MOUDs. Even for patients who reached a clinician, multiple scheduling attempts were typically required—appointments were granted on the first attempt in less than one-half of completed calls. Recently, SAMHSA launched FindTreatment.gov^42^ to connect patients to treatment clinicians; however, it is not clear whether the clinician list is different from the previous publicly available databases from the agency, and the website does not have a searchable option for clinicians who are willing to see pregnant women. SAMHSA could consider efforts to update or audit publicly available lists of clinicians to ensure their accuracy and provide the ability to search for clinicians willing to treat pregnant women.

Our study revealed that cost may be a significant barrier to MOUD access for women of reproductive age, even when clinicians are locally available. Similar to Beetham et al,^43^ we found that the median cost for an initial visit to a buprenorphine-waivered prescriber was $250 excluding medication costs, likely exceeding many families’ ability to pay.^44^ Overall, one-fourth of buprenorphine prescribers and one-third of OTPs granted appointments only if patients agreed to pay cash. There was also substantial variation across states in clinician acceptance of insurance for treatment. State policies may explain some of this variation. For example, Tennessee’s Medicaid program did not cover methadone in OTPs during the study period.^45^ Other states have policies that encourage clinicians to accept insurance. For example, West Virginia law requires that “prior to directly billing a patient [for MOUD] a treatment program must receive either a rejection of prior authorization, rejection of a submitted claim, or a written denial from a patient’s insurer or West Virginia Medicaid denying coverage for such treatment.”^46^ In addition, programs must document that the patient has no insurance before requesting cash payment. Even with this provision, we found that more than 40% of West Virginia buprenorphine-waivered prescribers did not accept insurance.

As the opioid crisis affected more communities across the United States, state and federal policy makers focused attention on improving access to treatment for individuals with OUD, including pregnant women. In our study sample, 4 states (Kentucky, Missouri, Tennessee, and West Virginia) had statewide policies prioritizing pregnant women for substance use treatment^47^; however, we found that, even in these states, treatment access for pregnant women was variable. Furthermore, while recent federal legislation focused specific provisions on pregnant women with OUD, these programs were mostly limited in scope and funding.^48^ Our study suggests that there remains an urgent need to systematically improve access to MOUDs among all women, and in particular pregnant women. To address this need, policy makers may consider enhancing outreach and training for women’s health professionals to increase the numbers of MOUD clinicians willing to accept pregnant women and explore mechanisms to incentivize clinicians to treat pregnant women with OUD.

### Limitations

Our study has several limitations. First, our study occurred in 10 selected US states and may not be generalizable to the rest of the nation. However, the states that we studied reflect 26% of the US population with a wide range of opioid-related complications. Next, we used publicly available treatment locators from SAMHSA to simulate a real-world patient experience; however, buprenorphine-waivered prescribers may opt out of this list, and it is possible that clinicians who opt out may respond differently to pregnant patients attempting to obtain an appointment for treatment. Our study only included female patients attempting to obtain access to treatment and therefore may not be generalizable to male patients. Calls were made at the practice level, and practice size was not considered in our analysis, perhaps underestimating patient access. In our analysis of wait time, multiple comparisons may be associated with findings of statistical significance. Last, the low number of OTPs in some states may result in imprecise estimates of access to MOUDs.

## Conclusions

In this cross-sectional study with random assignment of clinicians and simulated-patient callers, pregnant women were less successful than nonpregnant women of reproductive age in obtaining appointments for treatment by buprenorphine-waivered prescribers, despite the known risk to the mother and developing fetus for untreated OUD. For both pregnant and nonpregnant women in any treatment setting, challenges with acceptance of insurance and common expectations for cash payment present potential substantial barriers to care. As policy makers strive to combat the ongoing opioid crisis, they should consider mechanisms to lower barriers to care for women of reproductive age with OUD, for whom effective treatments exist but routine access to such treatments may not.

## Notes

Supplement. **eAppendix 1.** Simulation Patient Script **eAppendix 2.** Standardized Data Collection Form **eAppendix 3.** Randomization Scheme **eAppendix 4.** R Code Used for Analysis **eFigure 1.** Flow Diagram of Caller Attempting to Schedule Appointments With Waivered Buprenorphine Prescribers **eFigure 2.** Flow Diagram of Caller Attempting to Schedule Appointments at Opioid Treatment Programs **eTable 1.** Probability of Nonpregnant and Pregnant Women Obtaining an Appointment With a Buprenorphine Prescriber in Regression Analysis Clustering by Providers **eTable 2.** Characteristics of Pregnant and Nonpregnant Callers Attempting to Access Treatment as Randomized **eTable 3.** Reasons for Inability to Contact Provider a) Overall, b) by State for Buprenorphine Providers and c) by State for OTPs **eTable 4.** Number of Call Attempts Where a) Provider Was Reached, b) Overall, and c) Median Calls **eTable 5.** Ability of Pregnant and Non-Pregnant Women to Obtain Appointment for Treatment Among Buprenorphine-Waivered Prescribers and Opioid Treatment Programs **eTable 6.** Acceptance of a) Medicaid or Private Insurance for Treatment Among Buprenorphine-Waivered Prescribers and b) Medicaid for Treatment Among Opioid Treatment Programs **eTable 7.** Difference Between Pregnant and Nonpregnant Caller Success Rates **eTable 8.** Out-of-Pocket Costs Among Pregnant and Nonpregnant Callers Obtaining Cash Appointments Among Buprenorphine Prescribers and Opioid Treatment Providers **eTable 9.** Wait Time Among Pregnant and Nonpregnant Callers Obtaining Appointments Among Buprenorphine Prescribers and Opioid Treatment Providers
