# Supplementary material for: Association of Pregnancy and Insurance Status With Treatment Access for Opioid Use Disorder
Source: JAMA Netw Open. 2020 Aug 14;3(8):e2013456. doi: 10.1001/jamanetworkopen.2020.13456 (PMC7428808; doi:10.1001/jamanetworkopen.2020.13456)
Supplement: Supplement. — eAppendix 1. Simulation Patient Script eAppendix 2. Standardized Data Collection Form eAppendix 3. Randomization Scheme eAppendix 4. R Code Used for Analysis eFigure 1. Flow Diagram of Caller Attempting to Schedule Appointments With Waivered Buprenorphine Prescribers eFigure 2. Flow Diagram of Caller Attempting to Schedule Appointments at Opioid Treatment Programs eTable 1. Probability of Nonpregnant and Pregnant Women Obtaining an Appointment With a Buprenorphine Prescriber in Regression Analysis Clustering by Providers eTable 2. Characteristics of Pregnant and Nonpregnant Callers Attempting to Access Treatment as Randomized eTable 3. Reasons for Inability to Contact Provider a) Overall, b) by State for Buprenorphine Providers and c) by State for OTPs eTable 4. Number of Call Attempts Where a) Provider Was Reached, b) Overall, and c) Median Calls eTable 5. Ability of Pregnant and Non-Pregnant Women to Obtain Appointment for Treatment Among Buprenorphine-Waivered Prescribers and Opioid Treatment Programs eTable 6. Acceptance of a) Medicaid or Private Insurance for Treatment Among Buprenorphine-Waivered Prescribers and b) Medicaid for Treatment Among Opioid Treatment Programs eTable 7. Difference Between Pregnant and Nonpregnant Caller Success Rates eTable 8. Out-of-Pocket Costs Among Pregnant and Nonpregnant Callers Obtaining Cash Appointments Among Buprenorphine Prescribers and Opioid Treatment Providers eTable 9. Wait Time Among Pregnant and Nonpregnant Callers Obtaining Appointments Among Buprenorphine Prescribers and Opioid Treatment Providers [file jamanetwopen-3-e2013456-s001.pdf]

Supplementary Online Content

Patrick SW, Richards MR, Dupont WD, et al. Association of pregnancy and insurance status with treatment access for opioid use disorder. *JAMA Netw Open.* 2020;3(8):e2013456. doi:10.1001/jamanetworkopen.2020.13456

eAppendix 1. Simulation Patient Script
eAppendix 2. Standardized Data Collection Form
eAppendix 3. Randomization Scheme
eAppendix 4. R Code Used for Analysis
eFigure 1. Flow Diagram of Caller Attempting to Schedule Appointments With Waivered Buprenorphine Prescribers
eFigure 2. Flow Diagram of Caller Attempting to Schedule Appointments at Opioid Treatment Programs
eTable 1. Probability of Nonpregnant and Pregnant Women Obtaining an Appointment With a Buprenorphine Prescriber in Regression Analysis Clustering by Providers
eTable 2. Characteristics of Pregnant and Nonpregnant Callers Attempting to Access Treatment as Randomized
eTable 3. Reasons for Inability to Contact Provider a) Overall, b) by State for Buprenorphine Providers and c) by State for OTPs
eTable 4. Number of Call Attempts Where a) Provider Was Reached, b) Overall, and c) Median Calls
eTable 5. Ability of Pregnant and Non-Pregnant Women to Obtain Appointment for Treatment Among Buprenorphine-Waivered Prescribers and Opioid Treatment Programs
eTable 6. Acceptance of a) Medicaid or Private Insurance for Treatment Among Buprenorphine-Waivered Prescribers and b) Medicaid for Treatment Among Opioid Treatment Programs
eTable 7. Difference Between Pregnant and Nonpregnant Caller Success Rates
eTable 8. Out-of-Pocket Costs Among Pregnant and Nonpregnant Callers Obtaining Cash Appointments Among Buprenorphine Prescribers and Opioid Treatment Providers
eTable 9. Wait Time Among Pregnant and Nonpregnant Callers Obtaining Appointments Among Buprenorphine Prescribers and Opioid Treatment Providers

This supplementary material has been provided by the authors to give readers additional information about their work.

eAppendix 1. Simulation Patient Script

1. **Hi, is this the clinic at <Clinic address>?**
   IF NOT, BUT STILL AN ADDICTION CLINIC: write down the new address.

2. **I'd like to make an appointment for addiction treatment.**

   IF PREGNANT: **... and I'm four months pregnant.**

3. A. IF WALK-IN ONLY: **When is the earliest I can be seen?**
   What days and times are the walk-in hours?

   B. IF *LISTED PROVIDER* IS NOT AVAILABLE AT THAT #: **Is there anyone there that treats addiction? I could see anyone.**

   C. IF CAN*NOT* GET ANY APPOINTMENT: **What is the problem, why can't I be seen?**

   D. IF THERE IS A WAITLIST: **How long until I can be seen?**

4. DO NOT VOLUNTEER UNTIL AFTER APPT IS MADE UNLESS YOU ARE ASKED, OR AFTER TOLD WALK-IN ONLY: **I'm on [Medicaid / PRIVATE NAME], do you take that insurance?** OR **I don't have any insurance, will you still see me?**

   ➔ IF ASKED WHICH SPECIFIC MEDICAID PLAN: **I don't have my card in front of me right now, but I'm pretty sure it says [UNDERWRITING INSURANCE NAME] on it.**

   ➔ IF NOT TAKE YOUR INSURANCE: **What if I just pay cash?**

   ➔ IF WILL TAKE CASH: **What does a treatment visit cost? (Will that be the same each time I come?)** OR **What does each treatment visit cost?**

5. CANCEL THE APPOINTMENT.

   A. GENERAL: **Oh wait. I just realized I can't come that day. Look, I'll have to check something and get back to you. Please take me off the calendar for now because that isn't a day I can do. Thank you and I'm sorry, I'll have to call back.**

   B. GENERAL: **Oh, man. You know what, I am not going to be able to make that appointment. I am going to have to call back. I'm really sorry about that.**

   C. GENERAL: **Well that's just great – I'm looking at my calendar and I realize there is no way. Look, I'm sorry but I can't make that day after all – I'll have to call back.**

   D. DEFAULTED TO CASH PAY: **So you said it was $XX a visit, right? [HESITATE] Yeah, I think you should take me off the schedule - I'm going to try calling this other thing I heard about.**

6. IF CALL IS TAKING UP TOO MUCH TIME:
   A. **I have an appointment I need to get to, I just needed to know___________________.**
   B. **I'm running late for an interview, I really need to go.**

© 2020 Patrick SW et al. *JAMA Network Open.*

<u>Questions about Drug Use</u>

Backstory if needed:

A. I got hooked after taking these prescription painkillers for a bad sprain.
B. I was having a lot of back pain at work, and got hooked on some prescription painkillers.

Type of addiction:

A. I've been using oxys for a while now and I'd like to get help.
B. I need help to get off oxys. I've been on them for a while.

Use of other substances:

A. Well, heroin's cheaper, so sometimes I use that, but I prefer pills if I can find them. I do *<u>not</u>* use needles.
B. I snort heroin sometimes 'cause it's easier to get, but no needles – at least I don't do that.

Benzodiazepines or other prescription drugs:

A. No, I don't use anything else.

Alcohol:

A. I actually don't really drink – I don't like alcohol much.
B. Drinking was never a problem with me. [I certainly have not been drinking since getting pregnant].

Symptoms:

A. Sometimes I get sweats and my stomach hurts when I don't have oxys, but right now I feel fine.
B. Right now I feel fine, but you know sometimes I get sweats or a crampy stomach when I don't get oxys.

Last use:

A. I took a pill this morning.
B. This morning was my last one.

Route:

A. I usually take a pill, but sometimes I snort it. I don't use needles.
B. Well, no needles, but I sometimes snort it. Normally I just take a pill.

Frequency of use:

A. I use every couple of days.
B. Every few days or so.

Dose:

A. Probably about 80mg of oxy now, for about 4 years. Occasionally a bag of heroin.
B. 80mg on a day that I'm using for about 3 years. Occasionally a bag of heroin.

Motivators:

A. My family won't let me live with them anymore if I don't get help.
B. I'm just tired of living like this.

Previous Treatment?

A. I have never tried.
B. No, it always seemed too hard, but now I feel ready.

Other medical problems:

A. "No, I'm pretty healthy. I get colds sometimes, especially if the weather is changing."

© 2020 Patrick SW et al. *JAMA Network Open.*

B. The thing is, I'm really pretty healthy except for this terrible habit. I get colds sometimes.

Are you experiencing any type of medical emergency? No

Do you currently feel like you are withdrawing? No

What services are you interested in? Residential or Outpatient? Outpatient

Where do you get your drugs? A friend / I'd rather not say.

Any history of mental health issues / diagnoses? No. (No diagnoses, no psychiatrists)

Questions about Pregnancy

How far along:
A. I'm like 16 weeks pregnant.
B. I'm pregnant about 4 months.

Past pregnancies:
A. **If *not* pregnant:** No, I've never been pregnant.
B. **If pregnant:** This is my first time pregnant.

Prenatal care:
A. Yes. [**Where?**] I'd rather not say.

Motivators:
A. I'm afraid of losing the baby if I don't get into treatment.
B. How can I be a good mom if I don't get into treatment?

Are you experiencing any type of medical emergency? No

Other Questions

Do you have a job? Not right now.

Where does insurance coverage come from? I'm on my husband's insurance.

Where does your husband work? I'd really rather not say right now. I just want to know if this is a place I can get an appointment to start treatment.

Any children? Any children under age 18? No.

© 2020 Patrick SW et al. *JAMA Network Open.*

eAppendix 2. Standardized Data Collection Form

1. APPTDISP: What was the final disposition of this case?
   - ◯ Can't be seen here (1)
   - ◯ Regular appointment (2)
   - ◯ Hypothetical appointment date (3)
   - ◯ Walk-in offered (4)
   - ◯ Unable to get past appointment system restrictions (5)

2. [IF APPTDISP = 4] Appointment date and time for walk in:
   *Capture SOONEST date and time that you could come in for walk-ins
   - ◯ Appointment date (MM/DD/YY):
   - ◯ Appointment time:

   2A. Do you have to be there by a certain time to definitely be seen that day?
   - ◯ Yes (1)
   - ◯ No (2)

   2B. If you're not seen on the same day, can you get on a waiting list for another day?
   - ◯ Yes (1)
   - ◯ No (2)

3. [IF APPTDISP = 2 OR 3] Appointment date and time:
   - ◯ Appointment date (MM/DD/YY): ____________________
   - ◯ Appointment time: _______________________________

4. Did you need to resort to cash in order to get the appointment?
   - ◯ Yes (1)
   - ◯ No (2)

5. [IF CASH] Were you given the <u>total</u> cost of the visit?
   - ◯ Yes, fixed cost (1)
   - ◯ No (2)
   - ◯ A range or approximate (3)

   5A. [IF FIXED COST] How much is the total cost of the visit?

   5B. [IF RANGE] How much is the total cost of the visit?
   Low end of range: ______________________
   High end of range: ______________________

6. [IF CASH] What additional information were you given about cost?
   - ◯ Cost of a single treatment appointment following intake (1)
   - ◯ Monthly cost of treatment (2)
   - ◯ NONE OF THE ABOVE (3)

© 2020 Patrick SW et al. *JAMA Network Open.*

◯ Other, specify (4) ______________________

7. Is the office located at [ADDRESS PROVIDED]?
   ◯ Yes (1)
   ◯ No (2)

8A. [IF NO] Enter the new address below:
   ◯ Street address: ____
   ◯ City, state, zip: ____

8. Please specify information offered about treatment during the call. Did clinic staff mention … [CHECK ALL]
   ◯ Tapering (1)
   ◯ Maintenance (2)
   ◯ Detox (3)
   ◯ NONE OF THE ABOVE (4)
   ◯ Other, specify (5) _______________________

9. Please specify information requested during the call. Did clinic staff ask … [CHECK ALL]
   ◯ … about insurance before offering an appointment?
   ◯ … about health concerns or conditions before offering an appointment?
   ◯ … patient's SSN?
   ◯ … for patient's private insurance or Medicaid number?
   ◯ … patient's address?
   ◯ … patient's phone?
   ◯ … patient's age/birth date?
   ◯ … patient's gender?
   ◯ … other physical characteristics, such as height or weight?
   ◯ … anything about medical records?
   ◯ … patient's email address?
   ◯ … patient's emergency contact?
   ◯ NONE OF THE ABOVE

10. [IF APPTDISP = 1] Please choose main reason why securing an appointment was not possible.
   ◯ Not accepting any new patients (1)
   ◯ Slots are full – waitlist only (2)
   ◯ Not taking any pregnant patients (3)
   ◯ Not taking any Medicaid patients (4)
   ◯ Not taking any new Medicaid patients (5)
   ◯ Not taking any uninsured patients (7)
   ◯ Other, specify (8) _______________________

11A. Were you given information (a referral) about where else to go?
   ◯ Yes (1)
   ◯ No (2)

© 2020 Patrick SW et al. *JAMA Network Open.*

11B. [IF YES] What information were you given about where else to go?
- ◯ Named clinic [SPECIFY] (1) ______________________
- ◯ Named doctor [SPECIFY] (2) ______________________
- ◯ Named Program (3)
- ◯ Named hospital (4)
- ◯ Address (5)
- ◯ Phone (6)
- ◯ Email (7)

11. [IF WAITLISTED] What did the clinic tell you about the waitlist?

12. [IF APPTDISP = 3 OR 5] Why was making this appointment uncertain/impossible? [CHECK ALL]
    - ◯ Receptionist doesn’t know if they accept your insurance type (1)
    - ◯ They can’t progress in the appointment system without more information (2)
    - ◯ Case or file needs to be evaluated (3)
    - ◯ Other, specify (5) ______________________
    - ◯ Need to register with PCP or hospital system (6)

13. NOTES: Please give an objective play-by-play description of what happened in this conversation.

© 2020 Patrick SW et al. *JAMA Network Open.*

eAppendix 3. Randomization Scheme

The number of providers varied from 222 (West Virginia) to 1,613 (Florida). In Florida, the only state with 1000+ providers, we randomly selected 1,100 and randomly assigned each to one of the four patient profiles. In Massachusetts, Michigan, North Carolina, and Washington, states with between 550 and 999 providers, we randomly assigned two distinct profiles to some providers and one profile to the others in such a way that a total of 1,100 profiles were assigned. In Kentucky, Tennessee, and Virginia, states with between 367 and 549 providers, we randomly assigned three distinct profiles to some physicians and two distinct profiles to the others in such a way that 1,100 profiles were assigned. For Missouri and West Virginia, states with fewer than 367 providers, each physician was assigned three distinct profiles. Because there are a limited number of OTPs in each state, and given that our preliminary data suggested little difference between Medicaid and private insurance acceptance in OTPs, all OTPs were assigned two profiles (pregnant with Medicaid versus not pregnant with Medicaid.

a) **Unique practices by state**

	OTPs	Buprenorphine Prescribers
FL	70	1613
KY	24	486
MA	72	933
MI	44	686
MO	16	331
NC	70	694
TN	15	487
VA	40	447
WA	25	676
WV	9	222

© 2020 Patrick SW et al. *JAMA Network Open.*

b) Randomization scheme

Patient Profiles
PM: pregnant with Medicaid
PP: Pregnant with private insurance
NM: Not pregnant with Medicaid
NP: Not pregnant with private insurance

Randomization of Profiles to Buprenorphine Prescribers

Randomization Schemes

A: One profile per provider
Permuted block randomization of 4 profiles: (PM) (PP) (NM) (NP)

B: Two profiles per provider
Permuted block randomization of the 12 ordered ways of selecting two distinct profiles

(PM, PP)	(PM, NM)	(PM, NP)
(PP, PM)	(PP, NM)	(PP, NP)
(NM, PM)	(NM, PP)	(NM, NP)
(NP, PM)	(NP, PP)	(NP, NM)

C: Three profiles per provider
Permuted block randomization of the 24 ordered ways of selecting three distinct profiles
(PP, NM, NP) (PP, NP, NM) (NP, PP, NM) (NP, NM, PP) (NM, PP, NP) (NM, NP, PP)
(PM, NP, NM) (PM, NM, NP) (NP, PM, NM) (NP, NM, PM) (NM, PM, NP) (NM, NP, PM)
(PM, PP, NP) (PM, NP, PP) (PP, PM, NP) (PP, NP, PM) (NP, PM, PP) (NP, PP, PM)
(PM, PP, NM) (PM, NM, PP) (PP, PM, NM)(PP, NM, PM) (NM, PM, PP) (NM, PP, PM)

Assigning Randomization Schemes to States
Let *n* be the number of providers in a state, *k* and *m* be the number of providers in that state called 2 and 3 times, respectively.
1100 < *n* (Florida): Randomly select 1100 providers and apply Scheme **A**
549 < *n* < 1000 (Massachusetts, Michigan, North Carolina and Washington):
Let *k* = 1100 – n and *m* = 0. Randomly select *k* of *n* providers and apply Scheme **B**. Apply Scheme **A** to the remaining *n* – *k* providers
366 < *n* < 550 (Kentucky, Tennessee and Virginia)
Let *m* = 1100 – 2*n*. Randomly select m of n providers and apply Scheme **C**. Apply Scheme **B** to the remaining *k* = *n* – *m* providers.
n < 367 (Missouri and West Virginia)
Apply Scheme **C** to all providers
Note that this algorithm assigned 1100 profiles to providers in all states except Missouri and West Virginia, and that the number of providers assigned 2 or 3 profiles was minimized. No provider was assigned the same profile more than once. All orders in which these profiles can be assigned are equally likely.

Randomization of Profiles to Opioid Treatment Programs

Each program was assigned both PM and NM profiles. The order of these assignments was determined by permuted block randomization. That is, each program was randomly assigned either (PM, NM) or (NM, PM).

© 2020 Patrick SW et al. *JAMA Network Open.*

c) **Random patient assignment by providers in each state.**

Buprenorphine Prescribers:

	FL	KY	MA	MI	MO	NC	TN	VA	WA	WV
# of Providers	1100	486	933	686	331	694	487	447	676	222
1 Profile	1100	0	766	272	0	288	0	0	252	0
2 Profiles	0	358	167	414	0	406	361	241	424	0
3 Profiles	0	128	0	0	331	0	126	206	0	222

OTPs:

All OTPs were assigned 2 profiles.

	FL	KY	MA	MI	MO	NC	TN	VA	WA	WV
# of Providers	70	24	72	44	16	70	15	40	25	9

© 2020 Patrick SW et al. *JAMA Network Open.*

eFigure 1. Flow Diagram of Caller Attempting to Schedule Appointments With Waivered Buprenorphine Prescribers

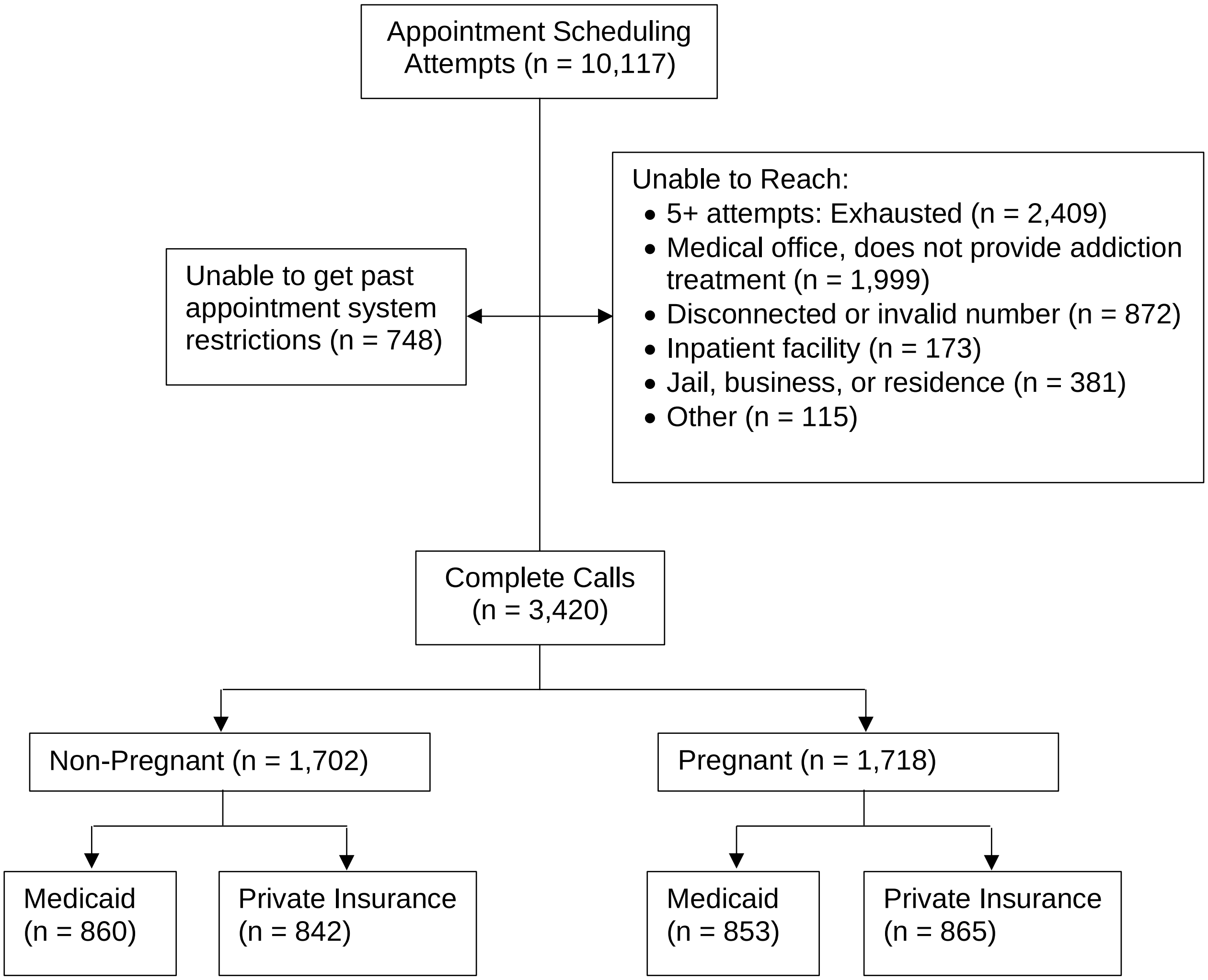

© 2020 Patrick SW et al. *JAMA Network Open.*

eFigure 2. Flow Diagram of Caller Attempting to Schedule Appointments at Opioid Treatment Programs

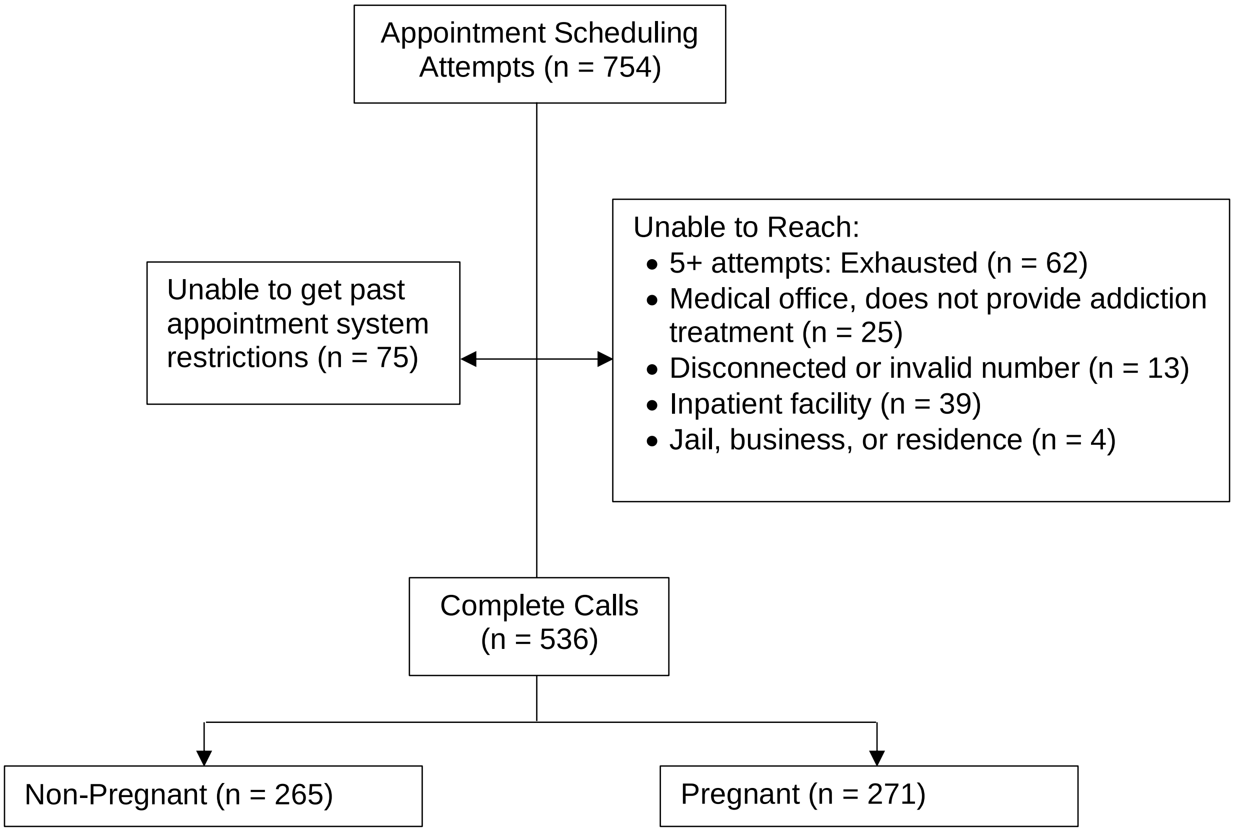

© 2020 Patrick SW et al. *JAMA Network Open.*

eTable 1. Probability of Nonpregnant and Pregnant Women Obtaining an Appointment With a Buprenorphine Prescriber in Regression Analysis Clustering by Providers

	Nonpregnant	Pregnant
	%<br>(95%CI)	%<br>(95%CI)
Buprenorphine Prescriber Acceptance	73.3%<br>(95% CI 71.4%-75.1%)	62.0%<br>(95%CI 59.6%-64.5%)

* mixed effects logistic regression model with random intercepts for providers used and predicted probabilities created.

© 2020 Patrick SW et al. *JAMA Network Open.*

eTable 2. Characteristics of Pregnant and Nonpregnant Callers Attempting to Access Treatment as Randomized

	**Buprenorphine Prescribers**			**Opioid Treatment Programs**		
	**Non-Pregnant** (N=5,037)	**Pregnant** (N=5,080)	**p-value**	**Non-Pregnant** (N=379)	**Pregnant** (N=375)	**p-value**
**Insurance**			0.87			
Public	50% (2,510)	50% (2,523)		100% (379)	100% (375)	
Private	50% (2,527)	50% (2,557)				
**Race**			0.10			0.15
Black	33% (1,671)	35% (1,775)		39% (146)	33% (125)	
Hispanic	11% (556)	10% (515)		9% (36)	8% (29)	
White	56% (2,810)	55% (2,790)		52% (197)	59% (221)	
**Age**			0.32			0.52
25	16% (796)	17% (879)		17% (65)	20% (76)	
26	17% (859)	16% (821)		17% (65)	16% (59)	
27	17% (847)	16% (837)		15% (56)	17% (65)	
28	17% (847)	17% (843)		16% (59)	17% (62)	
29	17% (862)	16% (834)		17% (66)	16% (61)	
30	16% (826)	17% (866)		18% (68)	14% (52)	
**State**			1.00			1.00
FL	11% (541)	11% (546)		18% (69)	19% (70)	
KY	11% (533)	11% (534)		6% (24)	6% (24)	
MA	11% (531)	10% (531)		19% (72)	19% (70)	
MI	11% (530)	10% (532)		11% (42)	11% (41)	
MO	9% (477)	9% (476)		4% (15)	4% (15)	
NC	10% (524)	11% (534)		18% (70)	18% (69)	
TN	11% (532)	11% (542)		4% (15)	4% (15)	
VA	10% (526)	10% (529)		10% (38)	10% (38)	
WA	10% (522)	10% (532)		7% (25)	7% (25)	
WV	6% (321)	6% (324)		2% (9)	2% (8)	

© 2020 Patrick SW et al. *JAMA Network Open.*

eTable 3. Reasons for Inability to Contact Provider a) Overall, b) by State for Buprenorphine Providers and c) by State for OTPs

a) Overall:

	**Buprenorphine Providers** # of Cases	**Opioid Treatment Programs** # of Cases
≥ 5 with no answer	2,409 (23.8%)	62 (8.2%)
Medical office, does not provide addiction treatment	1,999 (19.8%)	25 (3.3%)
Disconnected or invalid number	872 (8.6%)	13 (1.7%)
Inpatient facility	173 (1.7%)	39 (5.2%)
Jail, business, or residence	381 (3.8%)	4 (0.5%)
Other*	115 (1.1%)	0 (0.0%)

b) Buprenorphine Providers:

	FL	KY	MA	MI	MO	NC	TN	VA	WA	WV
≥ 5 with no answer	240 (22%)	230 (22%)	337 (32%)	211 (20%)	225 (24%)	244 (23%)	237 (22%)	280 (27%)	239 (23%)	166 (25%)
Medical office, does not provide addiction treatment	247 (23%)	219 (21%)	183 (17%)	225 (21%)	173 (18%)	174 (16%)	205 (19%)	213 (20%)	230 (22%)	130 (20%)
Disconnected or invalid number	104 (10%)	113 (11%)	72 (7%)	99 (9%)	77 (8%)	86 (8%)	102 (9%)	94 (9%)	57 (5%)	68 (11%)
Inpatient facility	17 (3%)	21 (2%)	46 (4%)	4 (0.4%)	12 (1%)	13 (1%)	11 (1%)	17 (2%)	20 (2%)	12 (2%)
Jail, business, or residence	42 (2%)	44 (4%)	40 (4%)	37 (3%)	28 (3%)	32 (3%)	27 (3%)	51 (5%)	63 (6%)	17 (3%)
Other*	5 (0.5%)	17 (2%)	8 (1%)	10 (1%)	14 (1%)	16 (2%)	14 (1%)	13 (1%)	11 (1%)	7 (1%)

c) OTPs:

	FL	KY	MA	MI	MO	NC	TN	VA	WA	WV
≥ 5 with no answer	12 (9%)	4 (8%)	11 (8%)	10 (12%)	2 (7%)	10 (7%)	0 (0%)	4 (5%)	8 (16%)	1 (6%)
Medical office, does not provide addiction treatment	4 (3%)	0 (0%)	9 (6%)	4 (5%)	2 (7%)	0 (0%)	0 (0%)	2 (3%)	4 (8%)	0 (0%)
Disconnected or invalid number	2 (1%)	0 (0%)	3 (2%)	3 (4%)	1 (3%)	1 (1%)	0 (0%)	2 (3%)	1 (2%)	0 (0%)
Inpatient facility	14 (10%)	0 (0%)	18 (13%)	1 (1%)	0 (0%)	3 (2%)	2 (7%)	1 (1%)	0 (0%)	0 (0%)
Jail, business, or residence	1 (1%)	2 (4%)	1 (1%)	0 (0%)	0 (0%)	0 (0%)	0 (0%)	0 (0%)	0 (0%)	0 (0%)
Other*	0 (0%)	0 (0%)	0 (0%)	0 (0%)	0 (0%)	0 (0%)	0 (0%)	0 (0%)	0 (0%)	0 (0%)

* Clinic out of state, hard refusal, problematic or suspicious case.

© 2020 Patrick SW et al. *JAMA Network Open.*

eTable 4. Number of Call Attempts Where a) Provider Was Reached, b) Overall, and c) Median Calls

a)

	Buprenorphine Prescribers			Opioid Treatment Programs		
Number of Attempts	Number of Cases	% of Cases	N Attempts	Number of Cases	% of Cases	N Attempts
1	1,665	48.7%	1,665	243	45.3%	243
2	875	25.6%	1,750	150	28.0%	300
3	413	12.1%	1,239	80	14.9%	240
4	255	7.5%	1,020	35	6.5%	140
5	147	4.3%	735	18	3.4%	90
6	59	1.7%	354	8	1.5%	48
7	5	0.1%	35	2	0.4%	14
8	1	0.0%	8	0	0.0%	0
Total	3,420	-	6,806	536		1,075

b)

	Buprenorphine Prescribers			Opioid Treatment Programs		
Number of Attempts	Number of Cases	% of Cases	N Attempts	Number of Cases	% of Cases	N Attempts
1	4,055	40.1%	4,055	334	44.3%	334
2	1,873	18.5%	3,746	181	24.0%	362
3	859	8.5%	2,577	94	12.5%	282
4	492	4.9%	1,968	48	6.4%	192
5	2,476	24.5%	12,380	73	9.7%	365
6	300	3.0%	1800	20	2.7%	120
7	55	0.5%	385	4	0.5%	28
8	6	0.1%	48	0	0.0%	0
9	1	0.0%	9	0	0.0%	0
Total	10,117		26,968	754		1,683

c)

	**Buprenorphine Prescribers** Median # of Attempts (IQR)	**Opioid Treatment Programs** Median # of Attempts (IQR)
Complete Cases	2 (1-3)	2 (1-3)
Entire Cohort	2 (1-5)	2 (1-3)

© 2020 Patrick SW et al. *JAMA Network Open.*

eTable 5. Ability of Pregnant and Non-Pregnant Women to Obtain Appointment for Treatment Among Buprenorphine-Waivered Prescribers and Opioid Treatment Programs

		Overall	FL	KY	MA	MI	MO	NC	TN	
**Buprenorphine Prescribers**										
Pregnant	**N**	1055/1718= 0.61	107/173= 0.62	90/187=0.48	64/113= 0.57	103/198= 0.52	128/183= 0.70	140/199= 0.70	139/215= 0.65	1
	**95%CI**	0.59-0.64	0.54-0.69	0.41-0.55	0.47-0.65	0.45-0.59	0.63-0.76	0.64-0.76	0.58-0.71	0
Non-Pregnant	**N**	1257/1702= 0.74	166/200= 0.83	127/178=0.71	88/120= 0.73	120/196= 0.61	140/179= 0.78	163/198= 0.82	160/207=0.77	1
	**95%CI**	0.72-0.76	0.77-0.88	0.64-0.77	0.65-0.80	0.54-0.68	0.72-0.84	0.76-0.87	0.71-0.82	0
**Opioid Treatment Programs**										
Pregnant	**N**	240/271= 0.89	45/51= 0.88	16/20= 0.80	33/38=0.87	17/23= 0.74	12/13= 0.92	56/59= 0.95	13/13= 1.00	2
	**95%CI**	0.84-0.92	0.77 – 0.94	0.58 – 0.92	0.73 – 0.94	0.54 – 0.87	0.67 – 1.00	0.86 – 0.98	0.77 – 1.00	0
Non-pregnant	**N**	237/265= 0.89	44/46= 0.96	19/20= 0.95	41/45= 0.91	15/19= 0.79	12/12= 1.00	53/56= 0.95	13/13= 1.00	2
	**95%CI**	0.85 – 0.93	0.85 – 0.99	0.76 – 1.00	0.79/0.96	0.57 – 0.91	0.76 – 1.00	0.85 – 0.98	0.77 – 1.00	0

		Overall	FL	KY	MA	MI	MO	NC	TN	
**Buprenorphine Prescribers**										
Pregnant	**N**	1055/1718= 0.61	107/173= 0.62	90/187=0.48	64/113= 0.57	103/198= 0.52	128/183= 0.70	140/199= 0.70	139/215= 0.65	1
	**95%CI**	0.59-0.64	0.54-0.69	0.41-0.55	0.47-0.65	0.45-0.59	0.63-0.76	0.64-0.76	0.58-0.71	0
Non-Pregnant	**N**	1257/1702= 0.74	166/200= 0.83	127/178=0.71	88/120= 0.73	120/196= 0.61	140/179= 0.78	163/198= 0.82	160/207=0.77	1
	**95%CI**	0.72-0.76	0.77-0.88	0.64-0.77	0.65-0.80	0.54-0.68	0.72-0.84	0.76-0.87	0.71-0.82	0
**Opioid Treatment Programs**										
Pregnant	**N**	240/271= 0.89	45/51= 0.88	16/20= 0.80	33/38=0.87	17/23= 0.74	12/13= 0.92	56/59= 0.95	13/13= 1.00	2
	**95%CI**	0.84-0.92	0.77 – 0.94	0.58 – 0.92	0.73 – 0.94	0.54 – 0.87	0.67 – 1.00	0.86 – 0.98	0.77 – 1.00	0
Non-pregnant	**N**	237/265= 0.89	44/46= 0.96	19/20= 0.95	41/45= 0.91	15/19= 0.79	12/12= 1.00	53/56= 0.95	13/13= 1.00	2
	**95%CI**	0.85 – 0.93	0.85 – 0.99	0.76 – 1.00	0.79/0.96	0.57 – 0.91	0.76 – 1.00	0.85 – 0.98	0.77 – 1.00	0

© 2020 Patrick SW et al. *JAMA Network Open.*

eTable 6. Acceptance of a) Medicaid or Private Insurance for Treatment Among Buprenorphine-Waivered Prescribers and b) Medicaid for Treatment Among Opioid Treatment Programs

		Overall	FL	KY	MA	MI	MO	NC	TN	VA	WA	WV
**Buprenorphine Prescribers**												
Medicaid	**N**	657/1713= 0.38	32/193= 0.17	72/171= 0.42	65/114= 0.57	52/197= 0.26	88/185= 0.48	103/202= 0.51	38/206= 0.18	61/161= 0.38	95/177= 0.54	51/107= 0.48
	**95%CI**	0.36 – 0.41	0.12 – 0.22	0.35 – 0.50	0.48 – 0.66	0.21 – 0.33	0.40 – 0.55	0.44 – 0.58	0.14 – 0.24	0.31 – 0.46	0.46 – 0.61	0.38 – 0.57
Private Insurance	**N**	761/1707= 0.45	55/180= 0.31	72/194 = 0.37	65/119= 0.55	94/197= 0.48	107/177= 0.60	111/195= 0.57	48/216= 0.22	65/159= 0.41	95/171= 0.56	49/99= 0.49
	**95%CI**	0.42 – 0.47	0.24 – 0.38	0.31 – 0.44	0.46 – 0.63	0.41 – 0.55	0.53 – 0.67	0.50 – 0.64	0.17 – 0.28	0.34 – 0.49	0.48 – 0.63	0.40 – 0.59
**Opioid Treatment Programs**												
Medicaid	**N**	303/536= 0.57	36/97= 0.37	18/40= 0.45	73/83= 0.88	20/42= 0.48	2/25= 0.08	73/115= 0.63	2/26= 0.08	40/59= 0.68	30/34= 0.88	9/15= 0.60
	**95%CI**	0.52 – 0.61	0.28 – 0.47	0.31 – 0.60	0.79 – 0.93	0.33 – 0.62	0.02 – 0.25	0.54 – 0.72	0.02 – 0.24	0.55 – 0.78	0.73 – 0.95	0.36 – 0.80

© 2020 Patrick SW et al. *JAMA Network Open.*

eTable 7. Difference Between Pregnant and Nonpregnant Caller Success Rates

	Buprenorphine Providers			Opioid Treatment Programs		
	Pregnant	Non-Pregnant	95% CI for Difference in Percentages	Pregnant	Non-Pregnant	95% CI for Difference in Percentages
Overall	61%	74%	9% - 16%	89%	89%	-5% - 7%
FL	62%	83%	12% - 31%	88%	96%	-5% - 20%
KY	48%	71%	4% - 30%	80%	95%	-10% - 40%
MA	57%	73%	4% - 30%	87%	91%	-12% - 21%
MI	52%	61%	-1% - 52%	74%	79%	-25% - 35%
MO	70%	78%	-1% - 18%	92%	100%	-14% - 30%
NC	70%	82%	3% - 21%	95%	95%	-9% - 8%
TN	65%	77%	4% - 22%	100%	100%	0% - 0%
VA	65%	66%	-9% - 13%	82%	74%	-32% - 16%
WA	66%	70%	-7% - 14%	94%	81%	-41% - 15%
WV	55%	72%	3% - 31%	100%	57%	-93% - 7%

© 2020 Patrick SW et al. *JAMA Network Open.*

eTable 8. Out-of-Pocket Costs Among Pregnant and Nonpregnant Callers Obtaining Cash Appointments Among Buprenorphine Prescribers and Opioid Treatment Providers

**Buprenorphine Prescribers**			
	**Non-Pregnant**<br>Min, Q1, Med, Q3, Max	**Pregnant**<br>Min, Q1, Med, Q3, Max	**p-value**
**Overall** (n = 847)	$0, $175, $250, $300, $915	$0, $150, $250, $300, $2000	0.10
**Medicaid** (n = 440)	$0, $175, $260, $325, $915	$15, $155, $250, $300, $2000	0.17
**Private** (n = 407)	$50, $175, $250, $300, $700	$0, $150, $250, $315, $850	0.30
**Opioid Treatment Programs**			
	**Non-Pregnant**<br>Min, Q1, Med, Q3, Max	**Pregnant**<br>Min, Q1, Med, Q3, Max	**p-value**
**Overall** (n = 160)	$6, $15, $26, $106, $250	$0, $15, $34, $125, $620	0.79

© 2020 Patrick SW et al. *JAMA Network Open.*

eTable 9. Wait Time Among Pregnant and Nonpregnant Callers Obtaining Appointments Among Buprenorphine Prescribers and Opioid Treatment Providers

	**Buprenorphine Prescribers**					
				**Non-Pregnant** Median (IQR)	**Pregnant** Median (IQR)	**p-value**
**Wait Time (days)**	**Overall** (n = 2,312)			3 (1, 7)	3 (1, 7)	0.43
		**Medicaid** (n = 1,123)		3 (1, 7)	3 (1, 7)	0.70
			**Without Cash** (n = 657)	4 (1, 7)	3 (1, 7)	0.46
			**With Cash** (n = 466)	3 (1, 7)	3 (1, 7)	0.78
		**Private** (n = 1,189)		3 (1, 7)	3 (1, 7)	0.46
			**Without Cash** (n = 761)	3 (1, 8)	3 (1, 8)	0.53
			**With Cash** (n = 428)	3 (1, 7)	3 (1, 7)	0.64
	**Opioid Treatment Programs**					
				**Non-Pregnant** Median (IQR)	**Pregnant** Median (IQR)	**p-value**
**Wait Time (days)**	**Overall** (n = 477)			2 (1, 6)	1 (1, 4)	0.05
		**Medicaid** (n = 477)		2 (1, 6)	1 (1, 4)	0.05
			**Without Cash** (n = 303)	2 (1, 6)	1 (1, 4)	0.02
			**With Cash** (n = 174)	2 (1, 5)	2 (1, 5)	0.88

© 2020 Patrick SW et al. *JAMA Network Open.*

eAppendix 4. R Code Used for Analysis

```
library(gtools)
library(tidyr)
library(Hmisc)
library(epitools)
library(ggplot2)
library(ggpubr)
library(lmtest)

#######################################
### Assigning Profiles to Providers ###
#######################################

##### Algorithm 1: Assigning 1 call per phone number #####
one_call <- function(df){
  ## Order by ClinicID
  df <- df[order(df$ClinicID),]

  ## Assign random uniform numbers
  df$rand <- runif(nrow(df))

  ## Order by rand
  df <- df[order(df$rand),]

  ## Assign profiles systematically
  profiles <- rep(c("A","B","C","D"),ceiling(nrow(df)/4))
  df$Profile <- profiles[1:nrow(df)]

  ## Add ClinicProfile column
  df$ClinicProfile <- df$Profile

  ## Add Ncalls column
  df$Ncalls <- rep(1,nrow(df))

  ## Add Order column
  df$Order <- rep(1,nrow(df))

  ## Keep only certain columns
  df <-
df[,c('ClinicID','Phone','ClinicProfile','Profile','Ncalls','Order','ProviderName','Clini
cName','ClinicType','Address','City','State','Zip','FIPS')]

  ## Return df
  df
}

##### Algorithm 2: Assigning 2 calls per phone number #####
two_calls <- function(df){
  ## Order by ClinicID
  twice <- df[order(df$ClinicID),]

  ## Assign random uniform numbers
  twice$rand <- runif(nrow(twice))

  ## Order by rand
  twice <- twice[order(twice$rand),]

  ## Assign profiles systematically
  profiles <-
rep(c("AB","AC","AD","BA","BC","BD","CA","CB","CD","DA","DB","DC"),ceiling(nrow(twice)/12
))
```

© 2020 Patrick SW et al. *JAMA Network Open.*

```
  twice$profile <- profiles[1:nrow(twice)]

  ## Split the profiles into two columns
  twice$profile1 <- regmatches(twice$profile, regexpr("+[[:upper:]]{1}",twice$profile))
  twice$profile2 <- regmatches(twice$profile, regexpr("[[:upper:]]{1}$",twice$profile))

  ## Add Ncalls column
  twice$Ncalls <- rep(2,nrow(twice))

  ## Convert to long format
  twice <- gather(twice, profile_id, Profile, profile1:profile2)

  ## Order by profile_id within ClinicID
  twice <- twice[order(twice$ClinicID,twice$profile_id),]

  ## Add ClinicProfile column
  twice$ClinicProfile <- twice$profile

  ## Assign order to profiles
  order <- rep(c(1,2),ceiling(nrow(twice)/2))
  twice$Order <- order[1:nrow(twice)]

  ## Keep only certain columns
  twice <-
twice[,c('ClinicID','Phone','ClinicProfile','Profile','Ncalls','Order','ProviderName','Cl
inicName','ClinicType','Address','City','State','Zip','FIPS')]

  ## Return twice
  twice
}

##### Algorithm 3: Assigning 3 calls per phone number #####
three_calls <- function(df){
  ## Order by ClinicID
  three <- df[order(df$ClinicID),]

  ## Assign random uniform numbers
  three$rand <- runif(nrow(three))

  ## Order by rand
  three <- three[order(three$rand),]

  ## Assign profiles systematically
  perms <- permutations(4,3,c("A","B","C","D"))
  groups <- paste(perms[,1],perms[,2],perms[,3],sep = " ")
  profiles <- rep(groups,ceiling(nrow(three)/24))
  three$profile <- profiles[1:nrow(three)]

  ## Split the profiles into three columns
  three$profile1 <- regmatches(three$profile, regexpr("+[[:upper:]]",three$profile))
  three$profile2 <- gsub(" ","",regmatches(three$profile,
regexpr("\\s{1}[[:upper:]]\\s{1}",three$profile)))
  three$profile3 <- regmatches(three$profile, regexpr("[[:upper:]]$",three$profile))

  ## Add Ncalls column
  three$Ncalls <- rep(3,nrow(three))

  ## Convert to long format
  three <- gather(three, profile_id, Profile, profile1:profile3)

  ## Order by profile_id within ClinicID
  three <- three[order(three$ClinicID,three$profile_id),]
```

© 2020 Patrick SW et al. *JAMA Network Open.*

```
  ## Add ClinicProfile column
  three$ClinicProfile <- gsub(" ","",three$profile)

  ## Assign order to profiles
  order <- rep(c(1,2,3),ceiling(nrow(three)/3))
  three$Order <- order[1:nrow(three)]

  ## Keep only certain columns
  three <-
three[,c('ClinicID','Phone','ClinicProfile','Profile','Ncalls','Order','ProviderName','Cl
inicName','ClinicType','Address','City','State','Zip','FIPS')]

  ## Return three
  three
}

##### Assigning profiles for buprenorphine providers #####
assign_profiles_bup <- function(df,seed){
  ## Set seed
  set.seed(seed)

  ## If >1100 phone numbers, sample 1100 phone numbers
  ## If <=1100 phone numbers, use all phone numbers
  if(nrow(df)>1100){
    state_sample <- df[sample(1:nrow(df),1100,replace = FALSE),]
  }else if(nrow(df)<=1100){
    state_sample <- df
  }

  ## Order by ClinicID
  state_sample <- state_sample[order(state_sample$ClinicID),]

  if(nrow(state_sample)>=1000){
    ## Assign each number to one profile
    final_sample <- one_call(state_sample)

    ## Order by ClinicID
    final_sample <- final_sample[order(final_sample$ClinicID),]

  }else if(nrow(state_sample)>=550 & nrow(state_sample)<1000){
    ## Find the number of phone numbers called once and the number of phone numbers
called twice
    num_twice <- 1100-nrow(state_sample)
    num_once <- nrow(state_sample)-num_twice

    ## Assign num_twice random phone numbers to group 2 and the remaining num_once to
group 1
    state_sample[sample(1:nrow(state_sample),num_twice),'group'] <- 2
    state_sample$group <- ifelse(is.na(state_sample$group),1,state_sample$group)

    ## Create two subsets of phone numbers to be called once and twice
    twice <- subset(state_sample,group==2)
    once <- subset(state_sample,group==1)

    final_twice <- two_calls(twice)
    final_once <- one_call(once)

    ## Combine numbers to be called once and twice and order by ClinicID
    combined <- rbind(final_twice,final_once)
    combined <- combined[order(combined$ClinicID),]
```

© 2020 Patrick SW et al. *JAMA Network Open.*

```
    # ## Order by ClinicID
    # final_twice <- final_twice[order(final_twice$ClinicID),]
    # final_once <- final_once[order(final_once$ClinicID),]
    #
    # ## List of numbers to be called once and twice
    # combined <- list(final_once,final_twice)

    final_sample <- combined

  }else if(nrow(state_sample)>=367 & nrow(state_sample)<=549){
    ## Find the number of phone numbers called three times and the number of phone
numbers called twice
    num_three <- 1100-2*nrow(state_sample)
    num_twice <- nrow(state_sample)-num_three

    ## Assign num_three random phone numbers to group 3 and the remaining num_twice to
group 2
    state_sample[sample(1:nrow(state_sample),num_three),'group'] <- 3
    state_sample$group <- ifelse(is.na(state_sample$group),2,state_sample$group)

    ## Create two subsets of phone numbers to be called twice and three times
    twice <- subset(state_sample,group==2)
    three <- subset(state_sample,group==3)

    ## Assign three profiles to each of the numbers in group 3
    final_three <- three_calls(three)
    ## Assign one profile to each of the numbers in group 2
    final_twice <- two_calls(twice)

    ## Combine numbers to be called twice and three times and order by ClinicID
    combined <- rbind(final_three,final_twice)
    combined <- combined[order(combined$ClinicID),]

    final_sample <- combined

  }else if(nrow(state_sample)<367){
    ## Assign three profiles to each number
    final_sample <- three_calls(state_sample)

    ## Order by ClinicID
    final_sample <- final_sample[order(final_sample$ClinicID),]

  }
  ## Return final_sample
  final_sample
}

##### Assigning profiles for OTPs #####
assign_profiles_otp <- function(df,seed){
  ## Set seed
  set.seed(seed)

  ## Order by ClinicID
  df <- df[order(df$ClinicID),]

  ## Assign random uniform numbers
  df$rand <- runif(nrow(df))

  ## Order by rand
  df <- df[order(df$rand),]

  ## Assign profiles systematically
```

© 2020 Patrick SW et al. *JAMA Network Open.*

```
  profiles <- rep(c("CD","DC"),ceiling(nrow(df)/2))
  df$profile <- profiles[1:nrow(df)]

  ## Split the profiles into two columns
  df$profile1 <- regmatches(df$profile, regexpr("+[[:upper:]]{1}",df$profile))
  df$profile2 <- regmatches(df$profile, regexpr("[[:upper:]]{1}$",df$profile))

  ## Add Ncalls column
  df$Ncalls <- rep(2,nrow(df))

  ## Convert to long format
  df <- gather(df, profile_id, Profile, profile1:profile2)

  ## Order by profile_id within ClinicID
  df <- df[order(df$ClinicID,df$profile_id),]

  ## Add ClinicProfile column
  df$ClinicProfile <- df$profile

  ## Assign order to profiles
  order <- rep(c(1,2),ceiling(nrow(df)/2))
  df$Order <- order[1:nrow(df)]

  ## Order by ClinicID
  df <- df[order(df$ClinicID),]

  ## Keep only certain columns
  df <-
df[,c('ClinicID','Phone','ClinicProfile','Profile','Ncalls','Order','ProviderName','Clini
cName','ClinicType','Address','City','State','Zip','FIPS')]

  ## Return df
  df
}


##############################
### Descriptive Statistics ###
##############################

## Descriptive Statistics for the Entire Cohort (eTable 3)
summaryM(InsuranceType+RaceProfile+PatAge+State~PatientPreg,data = final_bup, test =
TRUE, continuous = 10, overall = FALSE)
summaryM(InsuranceType+RaceProfile+PatAge+State~PatientPreg,data = final_otp, test =
TRUE, continuous = 10, overall = FALSE)

## Descriptive Statistics for Complete Calls (Table 1)
summaryM(InsuranceType+RaceProfile+PatAge+State~PatientPreg,data = complete_bup, test =
TRUE, continuous = 10, overall = FALSE)
summaryM(InsuranceType+RaceProfile+PatAge+State~PatientPreg,data = complete_otp, test =
TRUE, continuous = 10, overall = FALSE)


############################################################
### Reasons for Inability to Reach a Provider (eTable 1) ###
############################################################

table(final_bup$FinalDisposition)
table(final_bup$State,final_bup$FinalDisposition)
table(final_bup$APPTDISP)

table(final_otp$FinalDisposition)
```

© 2020 Patrick SW et al. *JAMA Network Open.*

```
table(final_otp$State,final_otp$FinalDisposition)
table(final_otp$APPTDISP)


##############################################
### Call Attempt Summary (eTable 2a and 2b) ###
##############################################

table(complete_bup$NumberofAttempts)
table(complete_otp$NumberofAttempts)

table(final_bup$NumberofAttempts)
table(final_otp$NumberofAttempts)

########################################################################
### Median and IQR of Attempts for Complete and All Calls (eTable 2c) ###
########################################################################

quantile(complete_bup$NumberofAttempts,probs = c(0.25,0.5,0.75))
quantile(complete_otp$NumberofAttempts,probs = c(0.25,0.5,0.75))

quantile(final_bup$NumberofAttempts,probs = c(0.25,0.5,0.75))
quantile(final_otp$NumberofAttempts,probs = c(0.25,0.5,0.75))


###################################################
### Relative Risks for Pregnant vs. Non-Pregnant ###
###################################################

preg_bup <- table(complete_bup$PatientPreg,complete_bup$apptMade)
riskratio(preg_bup,rev = "rows")
preg_otp <- table(complete_otp$PatientPreg,complete_otp$apptMade)
riskratio(preg_otp,rev = "rows")


########################################################################
### Percent Success for Pregnant and Non-Pregnant by State (Figure 1) ###
########################################################################

## Get the number of complete calls, number of successes, and percent success in each
state
by_state <- function(provider,preg){
  if(provider == "bup"){
    df <- complete_bup
  }else if(provider == "otp"){
    df <- complete_otp
  }
  complete <- subset(df,PatientPreg == preg)
  MadeAppt <- subset(df,apptMade==1&PatientPreg == preg)
  byState <- as.data.frame(cbind(table(complete$State),
                                 table(MadeAppt$State)
                                 ))
  colnames(byState) <- c("n","n_success")
  byState$Percent <- round(byState$n_success/byState$n*100,2)
  byState
}

byState_preg_bup <- by_state("bup",1)
byState_npreg_bup <- by_state("bup",2)
byState_preg_otp <- by_state("otp",1)
byState_npreg_otp <- by_state("otp",2)
```

© 2020 Patrick SW et al. *JAMA Network Open.*

```
    ## Add overall
    overall_preg_bup <- c(nrow(subset(complete_bup,PatientPreg ==
1)),nrow(subset(complete_bup,PatientPreg == 1&apptMade == 1)))
    overall_npreg_bup <- c(nrow(subset(complete_bup,PatientPreg ==
2)),nrow(subset(complete_bup,PatientPreg == 2&apptMade == 1)))
    overall_bup <- as.data.frame(rbind(overall_preg_bup,overall_npreg_bup))
    colnames(overall_bup) <- c("n","n_success")
    overall_bup$Percent <- round(overall_bup$n_success/overall_bup$n*100,2)
    byState_preg_bup <- as.data.frame(rbind(byState_preg_bup,overall_bup[1,]))
    rownames(byState_preg_bup) <- c(rownames(byState_preg_bup[1:10,]),"Overall")
    byState_npreg_bup <- as.data.frame(rbind(byState_npreg_bup,overall_bup[2,]))
    rownames(byState_npreg_bup) <- c(rownames(byState_npreg_bup[1:10,]),"Overall")

    overall_preg_otp <- c(nrow(subset(complete_otp,PatientPreg ==
1)),nrow(subset(complete_otp,PatientPreg == 1&apptMade == 1)))
    overall_npreg_otp <- c(nrow(subset(complete_otp,PatientPreg ==
2)),nrow(subset(complete_otp,PatientPreg == 2&apptMade == 1)))
    overall_otp <- as.data.frame(rbind(overall_preg_otp,overall_npreg_otp))
    colnames(overall_otp) <- c("n","n_success")
    overall_otp$Percent <- round(overall_otp$n_success/overall_otp$n*100,2)
    byState_preg_otp <- as.data.frame(rbind(byState_preg_otp,overall_otp[1,]))
    rownames(byState_preg_otp) <- c(rownames(byState_preg_otp[1:10,]),"Overall")
    byState_npreg_otp <- as.data.frame(rbind(byState_npreg_otp,overall_otp[2,]))
    rownames(byState_npreg_otp) <- c(rownames(byState_npreg_otp[1:10,]),"Overall")

## Percent Success Confidence Intervals
preg_bup_ci <- binconf(byState_preg_bup$n_success,byState_preg_bup$n,include.x =
TRUE,include.n = TRUE,return.df = TRUE)
rownames(preg_bup_ci) <- rownames(byState_preg_bup)
npreg_bup_ci <- binconf(byState_npreg_bup$n_success,byState_npreg_bup$n,include.x =
TRUE,include.n = TRUE,return.df = TRUE)
rownames(npreg_bup_ci) <- rownames(byState_npreg_bup)
preg_otp_ci <- binconf(byState_preg_otp$n_success,byState_preg_otp$n,include.x =
TRUE,include.n = TRUE,return.df = TRUE)
rownames(preg_otp_ci) <- rownames(byState_preg_otp)
npreg_otp_ci <- binconf(byState_npreg_otp$n_success,byState_npreg_otp$n,include.x =
TRUE,include.n = TRUE,return.df = TRUE)
rownames(npreg_otp_ci) <- rownames(byState_npreg_otp)

## Table at the bottom of Figure 1
preg_bup_ci$CI <- paste(round(preg_bup_ci$Lower,2),round(preg_bup_ci$Upper,2),sep = "-")
npreg_bup_ci$CI <- paste(round(npreg_bup_ci$Lower,2),round(npreg_bup_ci$Upper,2),sep = "-
")
preg_bup_ci$PointEst <- round(preg_bup_ci$PointEst,2)
npreg_bup_ci$PointEst <- round(npreg_bup_ci$PointEst,2)
bup <- rbind(t(preg_bup_ci),t(npreg_bup_ci))
bup <- bup[-c(1,2,4,5,7,8,10,11),]
rownames(bup) <- c("Pregnant Point Est.","Pregnant 95% CI","Not Pregnant Point Est.","Not
Pregnant 95% CI")

preg_otp_ci$CI <- paste(round(preg_otp_ci$Lower,2),round(preg_otp_ci$Upper,2),sep = "-")
npreg_otp_ci$CI <- paste(round(npreg_otp_ci$Lower,2),round(npreg_otp_ci$Upper,2),sep = "-
")
preg_otp_ci$PointEst <- round(preg_otp_ci$PointEst,2)
npreg_otp_ci$PointEst <- round(npreg_otp_ci$PointEst,2)
otp <- rbind(t(preg_otp_ci),t(npreg_otp_ci))
otp <- otp[-c(1,2,4,5,7,8,10,11),]
rownames(otp) <- c("Pregnant Point Est.","Pregnant 95% CI","Not Pregnant Point Est.","Not
Pregnant 95% CI")

## State Dot Plots by Pregnancy Status Including Wilson CIs
add_cis <- function(df,ci){
```

© 2020 Patrick SW et al. *JAMA Network Open.*

```
  df$lb <- ci$Lower*100
  df$ub <- ci$Upper*100
  df
}

byState_preg_bup <- add_cis(byState_preg_bup,preg_bup_ci)
byState_npreg_bup <- add_cis(byState_npreg_bup,npreg_bup_ci)
byState_preg_otp <- add_cis(byState_preg_otp,preg_otp_ci)
byState_npreg_otp <- add_cis(byState_npreg_otp,npreg_otp_ci)

by_state_dot_plot <- function(p,n,title){
  bystate_preg <- rbind(p,n)
  bystate_preg$state <- rownames(bystate_preg)
  bystate_preg$state <- gsub("\\d","",bystate_preg$state)
  bystate_preg$preg <- c(rep("Pregnant",11),rep("Not Pregnant ",11))

  bystate_preg$state <- factor(bystate_preg$state,
                               levels =
c("Overall","FL","KY","MA","MI","MO","NC","TN","VA","WA","WV"),
                               labels =
c("Overall","FL","KY","MA","MI","MO","NC","TN","VA","WA","WV"))

  state_preg <- ggplot(bystate_preg,aes(x = state,y = Percent,color = preg)) +
    geom_point(size = 4) +
    geom_errorbar(aes(ymin = lb, ymax = ub,width = .2)) +
    ylim(25,101) +
    ggtitle(title) +
    theme(legend.title = element_blank()) +
    xlab("State") +
    ylab("Percent Success")
  state_preg
}

bup_dot <- by_state_dot_plot(byState_preg_bup,byState_npreg_bup,"Buprenorphine
Providers")
otp_dot <- by_state_dot_plot(byState_preg_otp,byState_npreg_otp,"Opioid Treatment
Programs")

ggarrange(bup_dot,otp_dot,ncol = 2,common.legend = TRUE,legend = "bottom")


#######################################################
### Testing Appointment Access Variability by State ###
#######################################################

## Appointment access for pregnant callers by state
preg_bup <- subset(complete_bup,PatientPreg == 1)

mod1 <- glm(apptMade ~ State, data = preg_bup, family = "binomial")
mod2 <- glm(apptMade ~ 1, data = preg_bup, family = "binomial")

lrtest(mod1,mod2)

## Appointment access for non-pregnant callers by state
npreg_bup <- subset(complete_bup,PatientPreg == 2)

mod1.2 <- glm(apptMade ~ State, data = npreg_bup, family = "binomial")
mod2.2 <- glm(apptMade ~ 1, data = npreg_bup, family = "binomial")

lrtest(mod1.2,mod2.2)

## Medicaid acceptance for bup providers by state
```

© 2020 Patrick SW et al. *JAMA Network Open.*

```
medicaid_bup <- subset(complete_bup,InsuranceType == 1)
medicaid_bup$medAccepted <- ifelse(medicaid_bup$payment == "public"&medicaid_bup$apptMade
== 1,1,0)

mod3 <- glm(medAccepted ~ State, data = medicaid_bup, family = "binomial")
mod4 <- glm(medAccepted ~ 1, data = medicaid_bup, family = "binomial")

lrtest(mod3,mod4)
chisq.test(table(medicaid_bup$State,medicaid_bup$medAccepted))

## Private insurance acceptance for bup providers by state
private_bup <- subset(complete_bup,InsuranceType == 2)
private_bup$insAccepted <- ifelse(private_bup$payment == "private"&private_bup$apptMade
== 1,1,0)

mod5 <- glm(insAccepted ~ State, data = private_bup, family = "binomial")
mod6 <- glm(insAccepted ~ 1, data = private_bup, family = "binomial")

lrtest(mod5,mod6)
chisq.test(table(private_bup$State,private_bup$insAccepted))

## Medicaid acceptance for OTPs by state
medicaid_otp <- subset(complete_otp,InsuranceType == 1)
medicaid_otp$medAccepted <- ifelse(medicaid_otp$payment == "public"&medicaid_otp$apptMade
== 1,1,0)

mod7 <- glm(medAccepted ~ State, data = medicaid_otp, family = "binomial")
mod8 <- glm(medAccepted ~ 1, data = medicaid_otp, family = "binomial")

lrtest(mod7,mod8)
chisq.test(table(medicaid_otp$State,medicaid_otp$medAccepted))


###################################################################
### Ability to Obtain an Appointment by Insurance Type (Table 2) ###
###################################################################

## Non-Pregnant Patients
nonpreg <- subset(complete_bup,PatientPreg==2)  ## Bup
nonpreg <- subset(complete_otp,PatientPreg==2)  ## OTP
table(nonpreg$InsuranceType,nonpreg$apptMade)
## Medicaid Patients
nonpreg_m <- subset(nonpreg,InsuranceType==1)
tab_np_m <- table(nonpreg_m$payment,nonpreg_m$apptMade)
## Private Patients
nonpreg_p <- subset(nonpreg,InsuranceType==2)
tab_np_p <- table(nonpreg_p$payment,nonpreg_p$apptMade)

## Pregnant Patients
preg <- subset(complete_bup,PatientPreg==1)  ## Bup
preg <- subset(complete_otp,PatientPreg==1)  ## OTP
table(preg$InsuranceType,preg$apptMade)
## Medicaid Patients
preg_m <- subset(preg,InsuranceType==1)
tab_p_m <- table(preg_m$payment,preg_m$apptMade)
## Private Patients
preg_p <- subset(preg,InsuranceType==2)
tab_p_p <- table(preg_p$payment,preg_p$apptMade)

## Table 2a, Chi-Square Tests
chiSqTest <- function(med,priv,group){
  if(group == "accepted"){
```

© 2020 Patrick SW et al. *JAMA Network Open.*

```
    med_accepted <- med[2,2]
    priv_accepted <- priv[2,2]
    med_rejected <- sum(med[1,1],med[1,2],med[2,1])
    priv_rejected <- sum(priv[1,1],priv[1,2],priv[2,1])
  }
  if(group == "cash"){
    med_accepted <- med[1,2]
    priv_accepted <- priv[1,2]
    med_rejected <- sum(med[1,1],med[2,2],med[2,1])
    priv_rejected <- sum(priv[1,1],priv[2,2],priv[2,1])
  }
  if(group == "rejected"){
    med_accepted <- sum(med[1,1],med[2,1])
    priv_accepted <- sum(priv[1,1],priv[2,1])
    med_rejected <- sum(med[2,2],med[1,2])
    priv_rejected <- sum(priv[2,2],priv[1,2])
  }
  c <- chisq.test(matrix(c(med_accepted,priv_accepted,med_rejected,priv_rejected),ncol =
2),correct = FALSE)
  p <-
prop.test(c(med_accepted,priv_accepted),c(sum(med_accepted,med_rejected),sum(priv_accepte
d,priv_rejected)),correct = FALSE)
  r <-
riskratio(as.table(matrix(c(med_accepted,priv_accepted,med_rejected,priv_rejected),ncol =
2)),rev = "columns")
  list(c,p,r)
}

chiSqTest(tab_p_m,tab_p_p,"accepted")
chiSqTest(tab_np_m,tab_np_p,"accepted")
chiSqTest(tab_p_m,tab_p_p,"cash")
chiSqTest(tab_np_m,tab_np_p,"cash")
chiSqTest(tab_p_m,tab_p_p,"rejected")
chiSqTest(tab_np_m,tab_np_p,"rejected")


###########################
### Out-of-Pocket Costs ###
###########################

## Minimum, maximum, and quartiles for cost for bup providers
bup_cost <- subset(final_bup,COSTFLAT != -4)
quantile(bup_cost$COSTFLAT)

## Minimum, maximum, and quartiles for cost for OTPs
otp_cost <- subset(final_otp,COSTFLAT != -4)
quantile(otp_cost$COSTFLAT)

## Make factor for pregnancy
bup_cost$preg <- factor(bup_cost$PatientPreg,levels = c(1,2),labels = c("Pregnant","Not
Pregnant"))
otp_cost$preg <- factor(otp_cost$PatientPreg,levels = c(1,2),labels = c("Pregnant","Not
Pregnant"))

## Function to get min, max, and quartiles for table
costTab <- function(preg,clinic){
  if(clinic == "bup"){
    df <- bup_cost
  }else if(clinic == "otp"){
    df <- otp_cost
  }
  if(preg == "no"){
```

© 2020 Patrick SW et al. *JAMA Network Open.*

```
    data <- subset(df,PatientPreg == 2)
  }else if(preg == "yes"){
    data <- subset(df,PatientPreg == 1)
  }
  n_overall <- nrow(df)
  n_medicaid <- nrow(subset(df,InsuranceType == 1))
  n_private <- nrow(subset(df,InsuranceType == 2))
  n <- c(n_overall,n_medicaid,n_private)
  cost_overall <- quantile(data$COSTFLAT,na.rm = TRUE)
  cost_medicaid <- quantile(subset(data,InsuranceType == 1)$COSTFLAT,na.rm = TRUE)
  cost_private <- quantile(subset(data,InsuranceType == 2)$COSTFLAT,na.rm = TRUE)
  cost <- as.data.frame(rbind(cost_overall,cost_medicaid,cost_private))
  cost <- cbind(n,cost)
  rownames(cost) <- c("Overall","Medicaid","Private")
  cost
}

costTab(preg = "no",clinic = "bup")
costTab(preg = "yes",clinic = "bup")
costTab(preg = "no",clinic = "otp")
costTab(preg = "yes",clinic = "otp")

## Function for significance tests for cost
rankSumTest <- function(clinic){
  if(clinic == "bup"){
    df <- bup_cost
  }else if(clinic == "otp"){
    df <- otp_cost
  }
  preg <- subset(df,PatientPreg == 1)
  nonpreg <- subset(df,PatientPreg == 2)
  preg_medicaid <- subset(preg,InsuranceType == 1)
  preg_private <- subset(preg,InsuranceType == 2)
  nonpreg_medicaid <- subset(nonpreg,InsuranceType ==1)
  nonpreg_private <- subset(nonpreg,InsuranceType == 2)
  p1 <- wilcox.test(nonpreg$COSTFLAT,preg$COSTFLAT,correct = FALSE)$p.value
  p2 <- wilcox.test(nonpreg_medicaid$COSTFLAT,preg_medicaid$COSTFLAT,correct =
FALSE)$p.value
  if(clinic == "bup"){
    p3 <- wilcox.test(nonpreg_private$COSTFLAT,preg_private$COSTFLAT,correct =
FALSE)$p.value
  }
  if(clinic == "bup"){
    p_values <- c(p1,p2,p3)
    names(p_values) <- c("Overall","Medicaid","Private")
  }else if(clinic == "otp"){
    p_values <- c(p1,p2)
    names(p_values) <- c("Overall","Medicaid")
  }
  p_values
}

bup_p_values <- rankSumTest("bup")
otp_p_values <- rankSumTest("otp")


#################
### Wait Time ###
#################

## Restrict to only those who were able to make an appointment
MadeAppt_bup <- subset(complete_bup,apptMade==1)
```

© 2020 Patrick SW et al. *JAMA Network Open.*

```
MadeAppt_otp <- subset(complete_otp,apptMade==1)

## Make factor for pregnancy
MadeAppt_bup$preg <- factor(MadeAppt_bup$PatientPreg,levels = c(1,2),labels =
c("Pregnant","Not Pregnant"))
MadeAppt_otp$preg <- factor(MadeAppt_otp$PatientPreg,levels = c(1,2),labels =
c("Pregnant","Not Pregnant"))

## Find wait time
MadeAppt_bup$apptDate <- as.Date(MadeAppt_bup$APPTDAT,format = "%m/%d/%y")
MadeAppt_bup$lastContact <- as.Date(MadeAppt_bup$LastContactDate,format = "%m/%d/%y")
MadeAppt_bup$waitTime <-
as.numeric(difftime(MadeAppt_bup$apptDate,MadeAppt_bup$lastContact, units = "days"))

MadeAppt_otp$apptDate <- as.Date(MadeAppt_otp$APPTDAT,format = "%m/%d/%y")
MadeAppt_otp$lastContact <- as.Date(MadeAppt_otp$LastContactDate,format = "%m/%d/%y")
MadeAppt_otp$waitTime <-
as.numeric(difftime(MadeAppt_otp$apptDate,MadeAppt_otp$lastContact, units = "days"))

## Function to get wait times for eTable 4
waitTab2 <- function(preg,clinic){
  if(clinic == "bup"){
    df <- MadeAppt_bup
  }else if(clinic == "otp"){
    df <- MadeAppt_otp
  }
  if(preg == "no"){
    data <- subset(df,PatientPreg == 2)
  }else if(preg == "yes"){
    data <- subset(df,PatientPreg == 1)
  }
  n_overall <- nrow(df)
  n_medicaid <- nrow(subset(df,InsuranceType == 1))
  n_medicaid_cash <- nrow(subset(df,InsuranceType == 1&payment == "cash"))
  n_medicaid_wo_cash <- nrow(subset(df,InsuranceType == 1&payment != "cash"))
  n_private <- nrow(subset(df,InsuranceType == 2))
  n_private_cash <- nrow(subset(df,InsuranceType == 2&payment == "cash"))
  n_private_wo_cash <- nrow(subset(df,InsuranceType == 2&payment != "cash"))
  n <-
c(n_overall,n_medicaid,n_medicaid_wo_cash,n_medicaid_cash,n_private,n_private_wo_cash,n_p
rivate_cash)
  waitTime_overall <- quantile(data$waitTime,c(0.25,0.5,0.75),na.rm = TRUE)
  waitTime_medicaid <- quantile(subset(data,InsuranceType ==
1)$waitTime,c(0.25,0.5,0.75),na.rm = TRUE)
  waitTime_medicaid_cash <- quantile(subset(data,InsuranceType == 1&payment ==
"cash")$waitTime,c(0.25,0.5,0.75),na.rm = TRUE)
  waitTime_medicaid_without_cash <- quantile(subset(data,InsuranceType == 1&payment !=
"cash")$waitTime,c(0.25,0.5,0.75),na.rm = TRUE)
  waitTime_private <- quantile(subset(data,InsuranceType ==
2)$waitTime,c(0.25,0.5,0.75),na.rm = TRUE)
  waitTime_private_cash <- quantile(subset(data,InsuranceType == 2&payment ==
"cash")$waitTime,c(0.25,0.5,0.75),na.rm = TRUE)
  waitTime_private_without_cash <- quantile(subset(data,InsuranceType == 2&payment !=
"cash")$waitTime,c(0.25,0.5,0.75),na.rm = TRUE)
  waitTime <-
as.data.frame(rbind(waitTime_overall,waitTime_medicaid,waitTime_medicaid_without_cash,wai
tTime_medicaid_cash,waitTime_private,waitTime_private_without_cash,waitTime_private_cash)
)
  waitTime <- cbind(n,waitTime)
  rownames(waitTime) <- c("Overall","Medicaid","Medicaid without Cash","Medicaid with
Cash","Private","Private without Cash","Private with Cash")
  waitTime
```

© 2020 Patrick SW et al. *JAMA Network Open.*

```
}

waitTab2(preg = "no",clinic = "bup")
waitTab2(preg = "yes",clinic = "bup")
waitTab2(preg = "no",clinic = "otp")
waitTab2(preg = "yes",clinic = "otp")

## Function for significance tests for wait time
rankSumTest <- function(clinic){
  if(clinic == "bup"){
    df <- MadeAppt_bup
  }else if(clinic == "otp"){
    df <- MadeAppt_otp
  }
  preg <- subset(df,PatientPreg == 1)
  nonpreg <- subset(df,PatientPreg == 2)
  preg_medicaid <- subset(preg,InsuranceType == 1)
  preg_medicaid_wo_cash <- subset(preg,InsuranceType == 1 & payment != "cash")
  preg_medicaid_with_cash <- subset(preg,InsuranceType == 1 & payment == "cash")
  preg_private <- subset(preg,InsuranceType == 2)
  preg_private_wo_cash <- subset(preg,InsuranceType == 2 & payment != "cash")
  preg_private_with_cash <- subset(preg,InsuranceType == 2 & payment == "cash")
  nonpreg_medicaid <- subset(nonpreg,InsuranceType ==1)
  nonpreg_medicaid_wo_cash <- subset(nonpreg,InsuranceType == 1 & payment != "cash")
  nonpreg_medicaid_with_cash <- subset(nonpreg,InsuranceType == 1 & payment == "cash")
  nonpreg_private <- subset(nonpreg,InsuranceType == 2)
  nonpreg_private_wo_cash <- subset(nonpreg,InsuranceType == 2 & payment != "cash")
  nonpreg_private_with_cash <- subset(nonpreg,InsuranceType == 2 & payment == "cash")
  p1 <- wilcox.test(nonpreg$waitTime,preg$waitTime,correct = FALSE)$p.value
  p2 <- wilcox.test(nonpreg_medicaid$waitTime,preg_medicaid$waitTime,correct =
FALSE)$p.value
  p3 <-
wilcox.test(nonpreg_medicaid_wo_cash$waitTime,preg_medicaid_wo_cash$waitTime,correct =
FALSE)$p.value
  p4 <-
wilcox.test(nonpreg_medicaid_with_cash$waitTime,preg_medicaid_with_cash$waitTime,correct
= FALSE)$p.value
  if(clinic == "bup"){
    p5 <- wilcox.test(nonpreg_private$waitTime,preg_private$waitTime,correct =
FALSE)$p.value
    p6 <-
wilcox.test(nonpreg_private_wo_cash$waitTime,preg_private_wo_cash$waitTime,correct =
FALSE)$p.value
    p7 <-
wilcox.test(nonpreg_private_with_cash$waitTime,preg_private_with_cash$waitTime,correct =
FALSE)$p.value
  }
  if(clinic == "bup"){
    p_values <- c(p1,p2,p3,p4,p5,p6,p7)
    names(p_values) <- c("Overall","Medicaid","Medicaid w/o Cash","Medicaid
w/Cash","Private","Private w/o Cash","Private w/Cash")
  }else if(clinic == "otp"){
    p_values <- c(p1,p2,p3,p4)
    names(p_values) <- c("Overall","Medicaid","Medicaid w/o Cash","Medicaid w/Cash")
  }
  p_values
}

bup_p_values <- rankSumTest("bup")
otp_p_values <- rankSumTest("otp")
```

© 2020 Patrick SW et al. *JAMA Network Open.*

```
##############################################################
### Medicaid/Private Insurance Acceptance by State (Figure 2) ###
##############################################################

## Get the number of complete calls, number of successes, and percent success in each
state
by_state_ins <- function(provider,ins){
  if(provider == "bup"){
    df <- complete_bup
  }else if(provider == "otp"){
    df <- complete_otp
  }
  complete <- subset(df,InsuranceType == ins)
  if(ins == 1){
    pay <- "public"
  }else if(ins == 2){
    pay <- "private"
  }
  InsAccepted <- subset(df,apptMade==1&payment == pay)
  byState <- as.data.frame(cbind(table(complete$State),
                                table(InsAccepted$State)
  ))
  colnames(byState) <- c("n","ins_accepted")
  byState$Percent <- round(byState$ins_accepted/byState$n*100,2)
  byState
}

byState_med_bup <- by_state_ins("bup",1)
byState_priv_bup <- by_state_ins("bup",2)
byState_med_otp <- by_state_ins("otp",1)

    ## Add overall
    overall_med_bup <- c(nrow(subset(complete_bup,InsuranceType ==
1)),nrow(subset(complete_bup,payment == "public"&apptMade == 1)))
    overall_priv_bup <- c(nrow(subset(complete_bup,InsuranceType ==
2)),nrow(subset(complete_bup,payment == "private"&apptMade == 1)))
    overall_bup <- as.data.frame(rbind(overall_med_bup,overall_priv_bup))
    colnames(overall_bup) <- c("n","ins_accepted")
    overall_bup$Percent <- round(overall_bup$ins_accepted/overall_bup$n*100,2)
    byState_med_bup <- as.data.frame(rbind(byState_med_bup,overall_bup[1,]))
    rownames(byState_med_bup) <- c(rownames(byState_med_bup[1:10,]),"Overall")
    byState_priv_bup <- as.data.frame(rbind(byState_priv_bup,overall_bup[2,]))
    rownames(byState_priv_bup) <- c(rownames(byState_priv_bup[1:10,]),"Overall")

    overall_med_otp <- c(nrow(subset(complete_otp,InsuranceType ==
1)),nrow(subset(complete_otp,payment == "public"&apptMade == 1)))
    overall_otp <- as.data.frame(t(overall_med_otp))
    colnames(overall_otp) <- c("n","ins_accepted")
    overall_otp$Percent <- round(overall_otp$ins_accepted/overall_otp$n*100,2)
    byState_med_otp <- as.data.frame(rbind(byState_med_otp,overall_otp[1,]))
    rownames(byState_med_otp) <- c(rownames(byState_med_otp[1:10,]),"Overall")

    ## Percent Success Confidence Intervals

    add_cis <- function(df,ci){
      df$lb <- ci$Lower*100
      df$ub <- ci$Upper*100
      df
    }

    med_bup_ci <- binconf(byState_med_bup$ins_accepted,byState_med_bup$n,include.x =
TRUE,include.n = TRUE,return.df = TRUE)
```

© 2020 Patrick SW et al. *JAMA Network Open.*

```
    rownames(med_bup_ci) <- rownames(byState_med_bup)
    priv_bup_ci <- binconf(byState_priv_bup$ins_accepted,byState_priv_bup$n,include.x =
TRUE,include.n = TRUE,return.df = TRUE)
    rownames(priv_bup_ci) <- rownames(byState_priv_bup)
    med_otp_ci <- binconf(byState_med_otp$ins_accepted,byState_med_otp$n,include.x =
TRUE,include.n = TRUE,return.df = TRUE)
    rownames(med_otp_ci) <- rownames(byState_med_otp)

    byState_med_bup <- add_cis(byState_med_bup,med_bup_ci)
    byState_priv_bup <- add_cis(byState_priv_bup,priv_bup_ci)
    byState_med_otp <- add_cis(byState_med_otp,med_otp_ci)

## State Dot Plots by Insurance Type Including Wilson CIs
by_state_plot_ins <- function(m,p=NULL,title){
  if(title=="Buprenorphine Providers"){
    bystate_ins <- rbind(m,p)
  }else{
    bystate_ins <- m
  }
  bystate_ins$state <- rownames(bystate_ins)
  bystate_ins$state <- gsub("\\d","",bystate_ins$state)
  bystate_ins$state <- factor(bystate_ins$state,
                              levels =
c("Overall","FL","KY","MA","MI","MO","NC","TN","VA","WA","WV"),
                              labels =
c("Overall","FL","KY","MA","MI","MO","NC","TN","VA","WA","WV"))
  if(title=="Buprenorphine Providers"){
    bystate_ins$ins <- c(rep("Medicaid",11),rep("Private Insurance ",11))
  }else{
    bystate_ins$ins <- rep("Medicaid",11)
  }
  state_preg <- ggplot(bystate_ins,aes(x = state,y = Percent,color = ins)) +
    geom_point(size = 4) +
    geom_errorbar(aes(ymin = lb, ymax = ub,width = .2)) +
    ylim(0,100) +
    ggtitle(title) +
    theme(legend.title = element_blank()) +
    xlab("State") +
    ylab("Percent Accepted") +
    scale_color_brewer(palette = "Dark2")
  state_preg
}


bup_state_ins <- by_state_plot_ins(m = byState_med_bup,p = byState_priv_bup,title =
"Buprenorphine Providers")
otp_state_ins <- by_state_plot_ins(m = byState_med_otp,title = "Opioid Treatment
Programs")

ggarrange(bup_state_ins,otp_state_ins,ncol = 2,common.legend = TRUE,legend = "bottom")
```

© 2020 Patrick SW et al. *JAMA Network Open.*
